# Soil and root microbiome analysis and isolation of plant growth-promoting bacteria from hybrid buffaloberry (*Shepherdia utahensis* ‘Torrey’) across three locations

**DOI:** 10.3389/fmicb.2024.1396064

**Published:** 2024-09-09

**Authors:** Ananta Raj Devkota, Ty Wilson, Amita Kaundal

**Affiliations:** Plants, Soils, and Climate, College of Agricultural and Applied Sciences, Utah State University, Logan, UT, United States

**Keywords:** buffaloberry, microbiome, PGPR, indole acetic acid (IAA), biostimulants and sustainable agriculture, *Pseudomonas*, ACC deaminase, *Stenotrophomonas*

## Abstract

The effects of climate change are becoming increasingly hazardous for our ecosystem. Climate resilient landscaping, which promotes the use of native plants, has the potential to simultaneously decrease the rate of climate change, enhance climate resilience, and combat biodiversity losses. Native plants and their associated microbiome form a holo-organism; interaction between plants and microbes is responsible for plants’ growth and proper functioning. In this study, we were interested in exploring the soil and root microbiome composition associated with *Shepherdia utahensis*, a drought hardy plant proposed for low water use landscaping, which is the hybrid between two native hardy shrubs of Utah, *S. rotudifolia* and *S. argentea.* The bulk soil, rhizosphere, root, and nodule samples of the hybrid *Shepherdia* plants were collected from three locations in Utah: the Logan Campus, the Greenville farm, and the Kaysville farm. The microbial diversity analysis was conducted, and plant growth-promoting bacteria were isolated and characterized from the rhizosphere. The results suggest no difference in alpha diversity between the locations; however, the beta diversity analysis suggests the bacterial community composition of bulk soil and nodule samples are different between the locations. The taxonomic classification suggests *Proteobacteria* and *Actinobacteriota* are the dominant species in bulk soil and rhizosphere, and *Actinobacteriota* is solely found in root and nodule samples. However, the composition of the bacterial community was different among the locations. There was a great diversity in the genus composition in bulk soil and rhizosphere samples among the locations; however, *Frankia* was the dominant genus in root and nodule samples. Fifty-nine different bacteria were isolated from the rhizosphere and tested for seven plant growth-promoting (PGP) traits, such as the ability to fix nitrogen, phosphates solubilization, protease activity, siderophore, Indole Acetic Acid (IAA) and catalase production, and ability to use ACC as nitrogen source. All the isolates produced some amount of IAA. Thirty-one showed at least four PGP traits and belonged to *Stenotrophomonas, Chryseobacterium, Massilia, Variovorax*, and *Pseudomonas*. We shortlisted 10 isolates that showed all seven PGP traits and will be tested for plant growth promotion.

## 1 Introduction

Climate change poses a severe threat to the functioning of the entire earth system (Higgins et al., 2023) and is challenging environmental sustainability (Anderson and Song, 2020). The changing climate may exceed the ability of certain plant species to adapt to adverse environments (Jump and Peñuelas, 2005). It’s crucial to note that climate change and its associated stresses directly impact agroecosystems and critical components of our global food security (Rodriguez and Durán, 2020). Understanding the interaction between plants and microbes and harnessing their benefits may provide innovative opportunities for improved plant growth, enhanced resilience during stress, and environmental restoration endeavors (Iqbal et al., 2023).

Recent studies on plant adaptation strategies to environmental changes suggest that plants exhibit a “cry for help,” wherein they can interact with some beneficial microbes in the soil to enhance their resilience and ability to adapt to changing conditions (Rolfe et al., 2019). A study on host-mediated microbiome engineering to improve drought tolerance in wheat suggested that plants can modify native microbiomes to adapt to stress conditions (Jochum et al., 2019). Such studies indicate that the microbiome of a plant can be transferred to other plants and used as inoculum to improve their growth under stress conditions (Woo and Pepe, 2018). Hence, microbes associated with plants from extreme environments could serve as important assets with stress-alleviating capabilities, as they are accustomed to the harsh environmental conditions in nature (Durán et al., 2019).

Utah, famous for its extreme weather, is facing a water shortage, which is a challenge to growers trying to maintain their landscapes, with a dire need for low water-use landscaping to help reduce water use in landscapes (Hilaire et al., 2008). Using native, drought-tolerant species in managed landscaping would not only reduce the water consumption for maintaining the landscape but also provide a native aesthetic and enhance biodiversity (Meyer et al., 2009). The genus *Shepherdia* contains *S. rotundifolia*, which is well adapted to extreme drought conditions, has an appealing appearance, and is a wise choice for landscaping (Sriladda et al., 2014). However, *S. rotundifolia* is slow-growing and challenging to establish in disturbed soils in nursery and urban landscapes, which is a matter of concern for the growers (Mee et al., 2003; Sriladda et al., 2016). Similarly, another species, *S. argentea*, which is found in the western part of North America, also has excellent adaptability from dry soil to wet soil (Chen et al., 2009; Mee et al., 2003) and is limited to riparian basins (Richer et al., 2003). However, poor adaptability and high mortality of native *Shepherdia* species in managed landscapes is a significant setback, possibly due to the lack of interaction with the microbes in their native conditions. An interspecific hybrid between *S. rotundifolia × S. argentea* possesses promising qualities suitable for low water-use landscaping and higher adaptability to wet conditions than roundleaf buffaloberry (Sriladda et al., 2016). *S. utahensis*, the hybrid buffaloberry, an actinorhizal plant that forms nodules with *Frankia* and fix nitrogen, possesses the appealing leaf characteristics of *S. rotundifolia* and looks attractive in maintained landscapes (Sriladda et al., 2016).

Adaptation of plants and their growth in specific habitats is significantly supported by the microbes associated with the plants (Theis et al., 2016). Root microbiome, one of the richest and the most diverse communities on the earth, mainly includes rhizosphere microbiota colonizing the immediate soil zone surrounding the plant root and endosphere microbiota colonizing the internal tissues of the roots (Bai et al., 2022; Pascale et al., 2020). Plants and the rhizosphere microbiome often have a mutualistic relationship; the secretion of exometabolites from the plant’s roots into the soil helps develop a carbon-rich niche for the rhizosphere microbiome and the members of the microbiome, in return, support plants by supplying the nutrients, suppressing the pathogens and modulating levels of phytohormones (Bulgarelli et al., 2012). These compounds attract microbes from the bulk soil to the rhizosphere. Hence, the root microbiome is dependent on the bulk soil and derived from the bulk soil microbes; bulk soil is credited as the primary seed bank and initial inoculum for the root microbiome (Bulgarelli et al., 2015). Zarraonaindia et al. (2015) found that the root microbiome is the subset of the bulk soil microbiome. The bacteria and fungi associated with the plant’s rhizosphere affect the host plant’s immunity, nutrient acquisition, stress tolerance, and pathogen abundance (Coats and Rumpho, 2014).

A study on plant restoration using mycorrhizal inoculum from some reference ecosystems, improved the establishment of plants by increasing the abundance of mycorrhizal fungi in the degraded ecosystem (Maltz and Treseder, 2015). Ecological restoration is much needed in the current scenario of changes in the environmental parameters due to global climate change (Hobbs and Cramer, 2008). Hence, developing techniques for resilient ecosystems is our priority (Valliere et al., 2020). The use of microbes in ecological restoration is cited in many studies (de-Bashan et al., 2012; Heneghan et al., 2008; Koziol et al., 2018). A study reported plant growth-promoting rhizobacteria from another native plant, *Ceanothus velutinus* (Ganesh et al., 2022). Recently, it was reported that inoculation of native soil from the *C. velutinus* to propagation mix enhances the cutting propagation and isolation of IAA-producing rhizobacteria and promotes growth in Arabidopsis (Ganesh et al., 2024). Such approaches of using indigenous soil microbial communities may be utilized in propagating the *Shepherdia* species in the managed landscapes; introducing microbial inoculums from disparate habitat and environmental conditions may not be adaptable to the local soil and environment conditions and the host plant (Maltz and Treseder, 2015). Though many studies have been conducted in recent years regarding the composition and diversity of microbes associated with crops, no studies could be found regarding the microbiology and microbiome composition of native *Shepherdia* species and their newly developed hybrid (*S. utahensis*) plant. Despite the attempts to establish *Shepherdia* species in managed landscapes, remarkably little is known about the dynamics and composition of their microbiomes. Hence, this study aims to reveal the microbiome associated with the root and root-associated soil of buffaloberry plants available in Utah, USA, as the functional integrity of the plants depends on the related microbiomes as per holobiont theory (Rosenberg et al., 2009).

The microbiome of the hybrid buffaloberry may harbor plant growth-promoting microbes for its resilience to extreme conditions and is an excellent resource for developing biostimulants or biofertilizers. Therefore, with this hypothesis, we aimed to investigate the diversity of microbiome associated with the bulk soil, rhizosphere soil, roots, and nodules of the hybrid buffaloberry and compare it among the three geographical locations within Utah, USA to see whether there is variation in the bacterial community composition based on location and isolated plant growth promoting bacteria from the rhizosphere. We also isolated plant growth-promoting bacteria from the rhizosphere to develop biofertilizers/ bio stimulants for sustainable agriculture.

## 2 Materials and methods

The collection and processing of the samples were done following the protocol given by McPherson et al. (2018), with slight modifications (Ganesh et al., 2022).

### 2.1 Collection and processing of soil, rhizosphere, and root

#### 2.1.1 Collection

For bulk soil collection, the top layer around the root, up to 30 cm depth, was collected in well-labeled zip lock bags and stored on the ice. For root and rhizosphere collection, 4–6 lateral roots from each plant were excised using the 70% ethanol sterilized pruning scissors and kept inside sterilized, well-labeled 35 ml tubes containing autoclaved phosphate buffer (6.33 g/L NaH_2_PO4, 8.5 g/L Na_2_HPO4 anhydrous, pH = 6.5, 200 μl/L Silwet L-77). The tubes were shaken vigorously for 2 min to release the rhizosphere from the surface of the roots, and the roots were transferred to a new labeled tube with 70% ethanol sterilized forceps. The tubes containing rhizosphere and roots were stored in the ice buckets. For nodules collection, the roots containing the nodules were collected and stored in the well-labeled tubes with Phosphate buffer in the same way that we collected roots.

The samples were collected from different places within Utah (Table 1). The samples of the hybrid buffaloberry from Greenville farm, USU, were collected on 21st October 2022, and those from the USU Campus and Kaysville farm, USU, were collected on 24th and 25th October 2022 respectively. The samples were collected in triplicate, i.e., samples were collected from three plants in each location as three replicates for each sample.

**TABLE 1:** Location of *S. utahnensis* (hybrid buffaloberry).

SN	Location	Coordinates
1	USU Logan campus	41°44′27′′ N 111°48′39′′ W
2	USU Greenville farm	41°44′16′′ N 111°48′39′′ W
3	USU Kaysville farm	41°1′21′′ N 111°56′1′′ W

#### 2.1.2 Processing the samples in laboratory

##### 2.1.2.1 Surface sterilization of roots and nodules

Surface sterilization of roots and nodules from the USU campus and Greenville farm was done on the same day after returning from the field. However, the samples collected from distant Kaysville were kept on ice overnight and processed the next day. The procedure involved adding 35 ml of 50% bleach + 0.01% Tween was added to the 50 ml tubes containing roots, and the tubes were shaken vigorously for 30–60 s. The bleach solution was then poured off, and the roots were washed with 70% ethanol by shaking for 30–60 s. This step was followed by pouring off the 70% ethanol and washing the roots with sterile (double distilled and autoclave) water 4–6 times by shaking. 100 μl of the last wash was plated on 1/4 NA to check the sterilization process. The roots were then blotted dry in autoclaved clean paper towels (one for each sample) and cut into small (5–10 mm) pieces using sterile scissors and forceps. Finally, the roots were placed in clean, labeled 15 ml tubes and stored at −80°C. Similarly, the nodules were separated individually and stored in sterilized, well-labeled 15 ml tubes at −80°C.

##### 2.1.2.2 Processing rhizosphere samples

The processing of the rhizosphere samples was conducted with utmost care and precision. Samples from the nearer locations, such as the Greenville farm and USU campus in Logan, were processed on the same day, ensuring immediate handling. A meticulous approach was taken for samples from distant locations - they were refrigerated overnight to maintain their integrity and processed the next day. The tubes containing the rhizosphere samples were gently shaken to resuspend the soil and collected in a newly labeled 50 ml tube strained through a sterile 100-μm-mesh cell strainer. The tubes were centrifuged at 3000×*g* for 5 min at room temperature, and the supernatant was discarded. The tubes with rhizosphere pellets were kept on ice, and 1.5 ml phosphate buffer (without surfactant) was added to each tube to resuspend the pellets. The suspended liquid was transferred to a newly labeled 2 ml microfuge tube and centrifuged at 13000 rpm for 2 min at room temperature. The supernatant was discarded, the tubes were drained on a clean paper towel for some time, and the pellet was divided into two tubes. One was stored at −20°C till further use for DNA Extraction, and the other tube was stored at 4°C for bacteria isolation.

##### 2.1.2.3 Processing bulk soil sample

Bulk soil samples were kept on ice until the rhizosphere and root samples were processed. Very coarse soil samples mixed with pebbles, root, and leaf litter were sieved. Each time, after sieving a sample, the sieves were thoroughly sprayed with 70% ethanol and pat-dried with autoclaved Paper towel. Using a clean and sterilized metal spatula, 2–3 gm of sieved soil sample was transferred to a well-labeled 2 ml microfuge tube and stored at −20°C till further use. Two cups of soil samples from each bulk soil sample were sent for complete nutrient analysis at the USU Analytical Laboratory.

### 2.2 DNA extraction

#### 2.2.1 Preparation of root samples for DNA extraction

The frozen root and nodule samples were removed from −80°C in liquid nitrogen and ground to powder using an autoclaved mortar and pestle, continually adding liquid nitrogen to keep the samples frozen. The ground tissue was transferred to a 1.5 ml microfuge tube using a clean, sterilized spatula/ disposable spatula without letting it thaw.

#### 2.2.2 Extraction of DNA from soil, rhizosphere, root, and nodule samples

150 mg of powdered root and nodule samples and 200 mg of the rhizosphere and bulk soil from each sample were used to extract DNA. E.Z.N.A Plant DNA Kit and E.Z.N.A Soil DNA Kit from Omega BIO-TEK were used to extract DNA from root and soil samples and stored at −20°C.

### 2.3 Sequencing the isolated DNA

The isolated DNA was sent to the Genomics facility, Center for Integrated Biosystems, USU, for 16S rRNA sequencing to identify bacterial communities associated with the root and soil of the buffaloberry species. V4 region of 16S rRNA was amplified for the DNA samples obtained from rhizosphere and bulk soil using the V4 variable region- forward primers - 515F (5′-GTGCCAGCMGCCGCGGTAA-3′) and reverse primer - 806R (5′-GGACTACHVHHHTWTCTAAT-3′) (Gkarmiri et al., 2017). However, V5-V7 region of 16S rRNA was amplified for the DNA samples obtained from the root and nodules with V5-V7 (for endophytes) specific primers 799-Forward (5′ AACMGGATTAGATACCCKG) and 1193-Reverse (3′ ACGTCATCCCCACCTTCC)^1^ (Ganesh et al., 2022). The volume of the amplification reaction was 25 μl; the composition was 13 μl of water, with 10 μl of Platinum Hot Start PCR Master Mix (ThermoFisher), 0.5 μl of 10 mM F/R primers, and 1 μl of 5 ng/μl of the DNA as a template. The polymerase chain reaction (PCR) was conducted using a DNA Engine DYAD Peltier Thermal Cycler (BIO-RAD), and the conditions followed were:


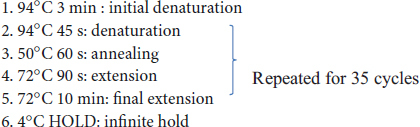


The PCR product was diluted fifty times, and secondary PCR was done to attach the indexes. The ingredients of the second PCR mixture were: 5 μl of Master mix, 2 μl (i5 index), 3 μl (i7 index), and 1 μl of the diluted PCR product. The PCR was run in a DNA engine dyad Peltier thermal cycler (BIO-RAD); the conditions followed were:


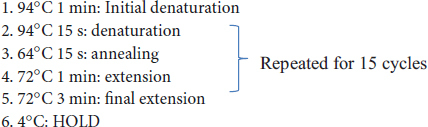


After attaching the indexes, the samples were cleaned with AMPureXP beads in the ratio 1:1 ratio. The PCR products were then quantified by fluorometry. The samples were pooled and sequenced on the MiSeq using a 2 × 250 paired-end sequencing (500 cycle Nano sequencing kit) (Illumina, San Diego, CA, USA).

### 2.4 Data analysis

The obtained 16S rRNA sequences were analyzed using the Qiime2 v 2023.5 (Caporaso et al., 2010).

For soil samples, fastq files were imported and then quality - controlled. Forward and reverse fastq reads were trimmed, filtered, and merged, and chimera were removed using divisive amplicon denoising algorithm (DADA2) with parameters: - -p-trim-left-f 10 - -p-trim-left-r 66 - -p-trunc-len-f 240 - -p-trunc-len-r 240 - -p-n-threads 24. Finally, the Amplicon Sequence Variants (ASVs) table was generated by mapping the pure reads to Naive Bayes Classifier trained Greengenes2-V4 database (gg_2022_10_backbone.v4.nb.qza) using Qiime feature-classifier classify-sklearn command.

For root samples, fastq files were imported and then quality controlled. Forward and reverse fastq reads were trimmed, filtered, and merged, and chimera were removed using DADA2 with parameters: - -p-trim-left-f 10 - -p-trim-left-r 10 - -p-trunc-len-f 240 - -p-trunc-len-r 240 - -p-n-threads 24. Finally, the ASV table was generated by mapping the pure reads to Naive Bayes Classifier trained Greengenes2-full length database (gg_2022_10_backbone_full_length.nb.qza) using Qiime feature-classifier classify-sklearn command.

The obtained ASV table was exported to R software for further alpha diversity and beta diversity analysis using the packages Phyloseq and Vegan. Alpha diversity was estimated by calculating Shannon indexes for measuring the evenness, i.e., the relative abundance of the species present in a sample, and Chao1 index for measuring the richness, i.e., for quantifying the number of species present. Principal Coordinate Analysis (PCoA) analysis using Bray Curtis dissimilarity matrices was performed to determine the bacterial community composition in the samples from different locations. Additionally, taxonomic classification at the phylum and genus level was done to identify the top ten most abundant phylum and genus in the samples from respective places. Statistical analyses for test of significance were done on the R and SAS statistical software; ggplot2 package in R software was used to draw plots.

### 2.5 Bacteria isolation and identification

The rhizosphere pellet of hybrid buffaloberry collected from the USU campus sites, which were stored at 4°C, were pooled into one tube. The pelleted rhizosphere samples were resuspended in double distilled and autoclaved water in a ratio of 1 gram of soil to 9.5 ml of water (Ganesh et al., 2022). The tubes were vortexed vigorously and serially diluted in a ratio of 1:10. 100 μl of the 10^–3^, 10^–4^, and 10^–5^ dilutions were plated on different media viz. 1/4 Nutrient Agar, Yeast Mannitol Agar (SIGMA-Life Science), Minimal M9 Media (BD Difco), and Actinomycete Isolation Agar (SIGMA-ALDRICH), DF minimal media with ACC as nitrogen source, and the plates were incubated at 28°C for 3–5 days depending on the bacterial colony growth (Supplementary Figure 1A). Upon the growth of colonies, based on the various visual characteristics like color, texture, transparency, size, consistency, and other distinct morphological traits, single colonies were selected and purified by multiple streak plating. The purified colonies were inoculated in Luria broth incubated at 28°C overnight; 750 μl of the bacterial solution and 750 μl of 50% glycerol were mixed in 2 ml tubes and stored at −80°C as glycerol stocks.

We conducted 16S rRNA gene amplification and sequencing to identify the isolated bacteria. For the 1.4 Kb 16S rRNA gene amplification, we ran PCR reactions using the 27F (V1 region- 5′-AGAGTTTGATCCTGGCTCAG-3′) as the forward primer and 1492R (V9 region- 5′-TACGGYTACCTTGTTACGACTT-3′) as the reverse primer using DreamTaq DNA polymerase. 40 μl PCR reactions were carried out by using 5 μl Dream Taq mix, 2 μl of each forward and reverse primer, 4 μl bacterial lysate, and 27 μl Nuclease-free water. To prepare bacterial lysate, a single colony of the bacterial isolates was picked using a sterile pipette tip, and swirled in 20 μl of nuclease-free water, and boiled at 95°C for 15 min to be used as a template for the PCR. The PCR product was sequenced by Sanger sequencing, and the obtained sequences were blasted against the 16S rRNA database on NCBI.

### 2.6 Characterization of bacterial isolates

The unique isolates based on morphological features (Supplementary Table 1) were tested for seven plant growth-promoting (PGP) – traits, such as the ability to produce siderophores, indole acetic acid (IAA), protease, and catalase, ability to solubilize phosphate and fix atmospheric nitrogen and to utilize ACC as nitrogen source. A single colony of each isolate was placed on a glass slide to test catalase activity, and 1–2 drops of hydrogen peroxide were added and mixed, with the appearance of bubbles marking positive catalase activity (Reiner, 2010). For phosphate solubilization screening, the isolates were patched on Pikovskaya medium and incubated at 28°C for 4 days; the presence of a clear halo around the patch indicated the isolates were capable of phosphate solubilization (Supplementary Figure 1C; Pikovskaya, 1948). The positive control used for phosphate solubilization was *Bacillus megaterium* ATCC14581 (Ganesh et al., 2024). The isolates were screened on CAS (chrome azurol S) agar (Millipore SIGMA) media for siderophore production (Arora and Verma, 2017; Schwyn and Neilands, 1987). The bacterial isolates were patched on CAS plates and incubated for 4 days at 28°C till the occurrence of yellow-orange halo around the bacteria indicative of siderophore production (Supplementary Figure 1B). *P. chlororaphis* O6 was used as a positive control for the siderophore production test (Ganesh et al., 2022). The isolates were patched in Skimmed milk agar plates and incubated at 28°C for 3–5 days; the appearance of a clear halo indicated the presence of protease activity (Hamza, 2017; Salarizadeh et al., 2014). *B. subtilis* was used as a positive control for protease activity (Supplementary Figure 1E; Ganesh et al., 2022). For the test of nitrogen-fixing ability, the bacterial isolates were tested on Norris Glucose Nitrogen-Free Medium; they were patched on the media plates and incubated at 28°C for 3–5 days till the presence of a clear halo around the colony, which was the positive indication of nitrogen fixation (Wafula et al., 2020). *Rhizobium leguminosarum* C6 was also patched on each plate as a positive control for nitrogen fixation (Supplementary Figure 1D; Ganesh et al., 2022). The bacterial isolates were also tested for IAA production; the isolates were cultured in 5 ml LB broth supplemented with 0.1% tryptophan. The cultures were then incubated at 28°C and continuous shaking at 180 rpm for 48 h. For control, a non-inoculated LB + tryptophane of equal volume was used. The bacterial growth was sedimented by centrifuging at 10000 rpm for 10 min, and 1 ml of supernatant of each isolate was mixed with 2 ml of Salkowski reagent and incubated at room temperature by wrapping with Aluminum foil for 25 min. The change in the color to pink indicated the IAA production and the intensity of the color increases with the increase in IAA production (Supplementary Figure 1G). 200 μl of each isolate’s supernatant + Salkowski reagent mix in triplicates was read at 530 nm in 96 well-round bottom plates in the Spectramax Microplate reader (Molecular Devices). To prepare the Salkowski reagent, 2 ml of 0.5 M FeCl_3_ was mixed in 49 ml of double distilled water. Then, 49 ml of 70% perchloric acid (SIGMAALDRICH) was carefully added to the mix in the chemical hood. An IAA standard curve was prepared by mixing 2 ml of Salkowski reagent and 1 ml of each 0, 5, 10, 20, 50, and 100 μg/ml concentrations of IAA solution, and the equation was used for the calculation of IAA produced by the bacterial isolates (Supplementary Figures 1F, 2; Gordon and Weber, 1951).

## 3 Results

### 3.1 Bulk soil from the campus is the richest in NPK and micro-nutrient content among the three locations

The soil analysis report provided by Utah State University Analytical Laboratories (USUAL) (Table 2) indicates that the soil sample from the Utah State University Logan campus has the highest organic matter content, and the soil sample from the Kaysville farm has the highest Salinity. The pH of the soil samples from all three locations is almost near the neutral pH value. The soil sample from the Logan campus is highest in nitrogen, phosphorus, and potassium, along with the micronutrients zinc, iron, copper, manganese, and sulfur.

**TABLE 2:** Bulk soil analysis result.

Location	pH	Salinity EC (ds/m)	P (mg/Kg)	K (mg/Kg)	N (mg/Kg)	Zn (mg/Kg)	Fe (mg/Kg)	Cu (mg/Kg)	Mn (mg/Kg)	S (mg/ Kg)	OM (%)
Greenville	7.9	0.65	14.5	106	6.56	2.35	7.74	0.77	4.46	4.1	2.9
Campus	7.7	0.77	65.4	439	20.4	6.65	44.1	4.95	5.6	9.6	6.1
Kaysville	7.7	2.02	9.45	231	5.3	1.76	9.09	1.37	7.39	19	2.9

### 3.2 Alpha diversity of bacterial community is similar in all cases except in the nodule samples

**FIGURE 1:**
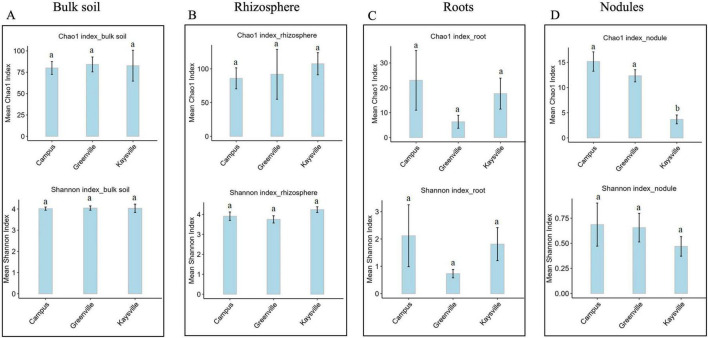
Alpha-diversity indices for Bacterial communities in panel **(A)** bulk soil, **(B)** rhizosphere, **(C)** root, **(D)** nodules of hybrid buffaloberry. Different lowercase letters represent significant differences (*P* ≤ 0.05) according to Tukey HSD.

We hypothesized that variations in micro-climate across distinct locations might influence the composition and abundance of bacterial communities. Nonetheless, the statistical analysis, specifically an ANOVA, indicates that there are no statistically significant differences (*p* < 0.05) observed in the species richness and evenness of the bulk soil, rhizosphere, and root bacterial community among the three locations, as assessed by the Chao1 index and Shannon index, respectively (Figure 1). However, in the nodule samples, there is a significant difference (*p* < 0.05) in the Chao 1 index but no difference in the Shannon index (Supplementary Tables 12, 14). The nodule samples from Kaysville have significantly lower Chao 1 indexes than those from Greenville and Campus. However, the Chao 1 indexes for Greenville and Campus are statistically similar (*p* < 0.05) to each other (Supplementary Table 13).

### 3.3 Bacterial community composition is different in bulk soil and nodule but not in root and rhizosphere

**FIGURE 2:**
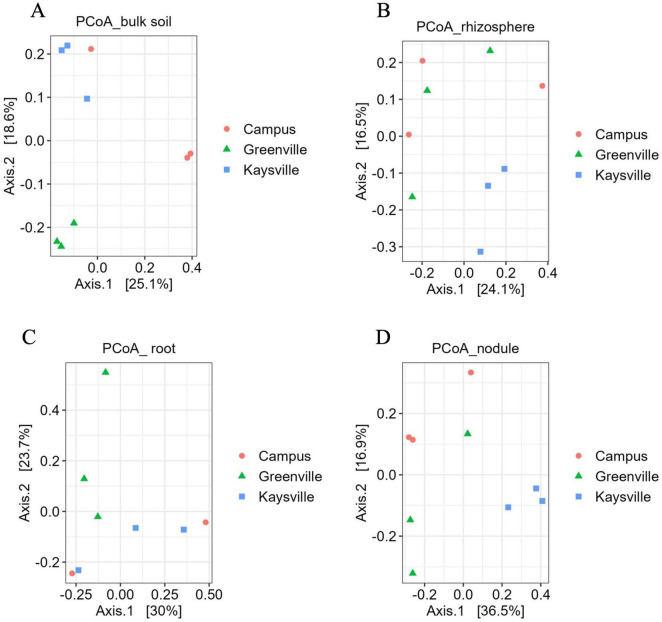
Principal coordinates analyses (PCoA) for bacterial communities in panel **(A)** bulk soil, **(B)** rhizosphere, **(C)** root, **(D)** nodules of hybrid buffaloberry using Bray-Curtis distance.

A β-diversity analysis based on PCoA was performed to compare the bacterial composition of the bulk soil, rhizosphere, root, and nodule samples of hybrid buffaloberry from the three locations (Figure 2). The PCoA plot is based on Bray-Curtis distances, which shows that the bacterial communities in the bulk soil of hybrid buffaloberry are separated based on the locations, which explains 43.7% (25.1 and 18.6%) of the overall variation. The samples from the three locations form three distinct clusters, which are separated from each other, suggesting a difference in the bacterial composition of the bulk soil among the three locations (Figure 2A). The bulk soil microbiome samples from Greenville in the PCoA plot are closer together than those from Kaysville and Campus, suggesting a higher degree of similarity among the samples from Greenville. However, the permutational multivariate analysis of variance analysis (PERMANOVA) performed using adonis2 indicates that there is a significant difference (*p* < 0.05) in bacterial composition among the samples from different locations (Supplementary Table 5).

Similarly, the bacterial communities in the nodules are also well separated based on the location, which explains 53.4% (36.5 and 16.9%) of the overall variation. However, the bacteria communities from the Greenville samples are seen to spread more; they are not mixed with the samples from the other two locations, which are in their respective clusters (Figure 2D). It suggests more variation in the bacterial communities within the Greenville samples than those from Kaysville and Campus, which are closer together, suggesting less variation within the samples. The PERMANOVA test also supports that there is a significant difference (*p* < 0.05) in the composition of bacterial communities among the samples from different locations (Supplementary Table 15).

In contrast, the bacterial communities of the rhizosphere and root samples from the three locations are dispersed in the PCoA plot, and all the bacterial communities from different locations are mixed and not separated (Figures 2B, C). It indicates that the bacterial communities of the rhizosphere and root samples of hybrid buffaloberry from different locations are not distinct from each other, and this is supported by the PERMANOVA test, which signifies that there is no significant difference (*p* > 0.05) in the bacterial composition among the samples from different locations (Supplementary Tables 8, 11).

### 3.4 *Proteobacteria* and *Actinobacteriota* are the dominant phyla in the bulk soil and rhizosphere however *Actinobacteriota* is the dominant phylum in the root and nodules from all locations

**FIGURE 3:**
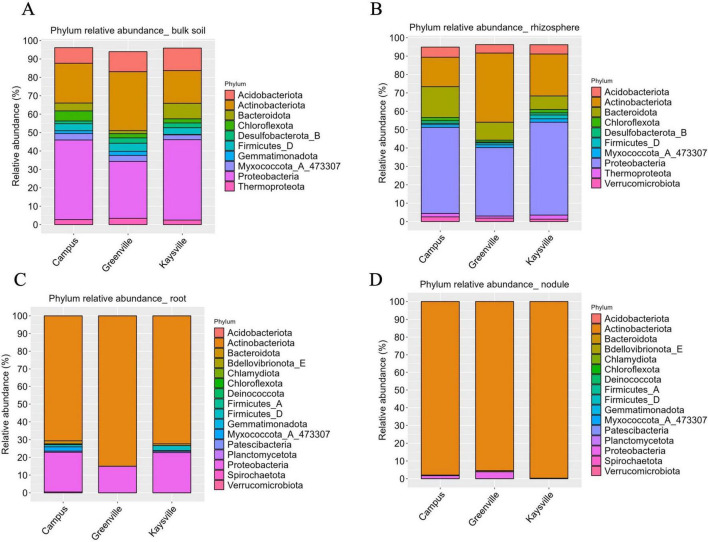
The relative abundances of the 10 most abundant phyla in panel **(A)** Bulk soil, **(B)** Rhizosphere, **(C)** Root, **(D)** Nodules of hybrid buffaloberry from the three different locations.

In the bulk soil samples of Kaysville and Campus, *Proteobacteria* is present in significantly higher abundance (*p* < 0.05). In contrast, in the Greenville samples, *Actinobacteriota* and *Proteobacter*ia have statistically similar (*p* < 0.05) abundance and are significantly higher (*p* < 0.05) than others (Figure 3A). *Proteobacteria* (30.95% in Greenville, 43.69% in Kaysville, and 43.24% in Campus), *Actinobacteriot*a (32.02% in Greenville, 17.78% in Kaysville, and 21.62% in the Campus), and *Acidobacteriota* (10.89% in Greenville, 12.23% in Kaysville, and 8.49% in the Campus) are the most dominant phyla in the bulk soil samples from all the three locations (Figure 3A). Additionally, notable contributions come from *Bacteroidota, Chloroflexota, Desulfobacterota, Myxococcota, Thermoproteota, Gemmatimonadota*, and *Firmicutes*, which exhibit substantial representation in bulk soil environments. *Bacteroidota* has a comparatively higher presence (8.45%) in the Kaysville samples than the Campus samples (4.27%) and the Greenville samples (1.63%). *Chloroflexota* has a higher abundance in the Campus samples (5.48%) than in Greenville (2.26%) and Kaysville (2.23%) (Figure 3A). Similarly, the rhizosphere samples follow the same pattern as bulk soil, where the presence of *Proteobacteria* is significantly higher (*p* < 0.05) in the samples from Kaysville (50.5%) and Campus (46.78%) (Figure 3B). The Greenville samples have significantly higher (*p* < 0.05) *Actinobacteriota* (37.63%) and *Proteobacteria* (37.2%), while their presence is statistically comparable (*p* < 0.05). *Actinobacteriota* (37.63%in Greenville, 22.83% in Kaysville, and 15.99% in Campus), *Proteobacteria* (50.5% in Kaysville, 46.78% in Campus and 37.2% in Greenville), *Bacteroidota* (9.74% in Greenville, 7.39% in Kaysville, and 16.79% in Campus), and *Acidobacteriota* (4.63% in Greenville, 5.06% in Kaysville, and 5.54% in Campus) are the top four phyla present in the rhizosphere samples of hybrid buffaloberry from all the three locations (Figure 3B). Other phyla in the rhizosphere samples are *Chloroflexota, Desulfobacterota, Myxococcota, Thermoproteota, Verrucomicrobiota*, and *Firmicutes* (Figure 3B).

Regarding the root and nodule samples, the phylum *Actinobacteriota* is present significantly higher (*p* < 0.05) in all three locations regarding their abundance (Figures 3C, D). *Actinobacteriota* is the most dominant phylum in the root samples of hybrid buffaloberry in all the locations, with 85% abundance in Greenville, 72.33% in Kaysville, and 70.64% in the Campus (Figure 3C). Among the other phyla in the root samples, *Proteobacteria* has a higher abundance: 14.97% in the Greenville samples, 22.71% in the Kaysville samples, and 22.45% in the Campus samples (Figure 3C). All locations exhibit traces of *Patescibacteria* (0.76% in Kaysville, 0.63% in Campus, and 0.03% in Greenville), while *Bacteroidota*, *Firmicute*s, and *Myxococcota* are also available phyla in both Kaysville and USU Campus. However, *Spirochaetota* (0.47%) is exclusively present on the USU Campus. Likewise, *Actinobacteriota* (99.71% in Kaysville, 97.98% in Campus, and 95.38% in Greenville) is the most dominant phylum in the nodule samples of hybrid buffaloberry from all three locations (Figure 3D). Other phyla are present but in smaller proportions. *Proteobacteria* is also present in all the locations, albeit in a much lower abundance of 3.87% in Greenville, 1.69% in Campus, and 0.29% in Kaysville. *Bacteroidota* is observed in both Kaysville and USU Campus samples, while *Patescibacteria* (0.11%) is exclusively present in Greenville samples. Furthermore, *Myxococcota* (0.29%) and *Chloroflexota* (0.01%) are specific to USU Campus samples (Figure 3D). A histogram (Supplementary Figure 3) describes a more comprehensive phylogenetic distribution of bulk, rhizosphere, roots, and nodules.

### 3.5 The genus classification is highly diverse in bulk soil and rhizosphere from all the locations however genus *Frankia* is dominant in the root and nodules from all the locations

**FIGURE 4:**
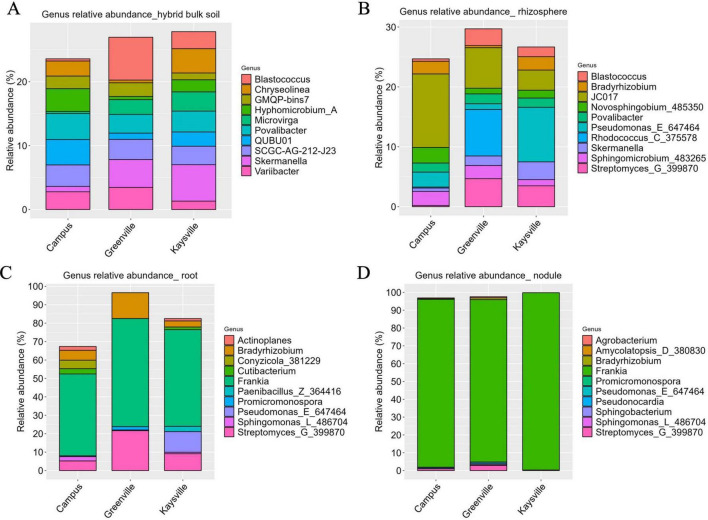
The relative abundances of the 10 most abundant genera in panel **(A)** Bulk soil, **(B)** Rhizosphere, **(C)** Root, **(D)** Nodules of hybrid buffaloberry from the three different locations.

There isn’t a single genus whose abundance stands out significantly higher than others in the bulk soil samples from all three locations. The genus *Blastococcus* (6.7%) was significantly higher (*p* < 0.05) in Greenville bulk soil samples whose presence was statistically similar (*p* < 0.05) to that of *Skermanella* (4.36%) and *Variibacter* (3.46%) (Figure 4A). The abundance of the genus *Skermanella* (5.7%) was significantly superior (*p* < 0.05) in Kaysville bulk soil samples, and it was statistically similar (*p* < 0.05) to *Chryseolinea* (3.81%) (Figure 4A). However, in the case of bulk soil samples from the Campus, no genus was present in significantly superior (*p* < 0.05) abundance (Figure 4A). The most abundant genera in the bulk soil samples from the Campus are *Povalibacter* (4.06%), *QUBU01*(3.98%), *Hyphomicrobium_A* (3.5%), SCGC-AG-212-J23 (3.36%), *Variibacter* (2.77%), *Chryseolinea* (2.35%), and GMQP-bins7(1.98%). Notably, *Blastococcus* (0.35%), *Skermanella* (0.84%), and *Microvirga* (0.32%) exhibit lower abundance in USU Campus samples as compared to the other two locations (Figure 4A). Similarly, the prevalence *of QUBU01*(3.98%) and *Hyphomicrobium_A* (3.5%) in USU Campus samples is comparatively higher than in Kaysville (2.23% and 1.89%, respectively) and Greenville (0.98% and 0.49% respectively) samples. This pattern is mirrored in the case of *Chryseolinea*, with Greenville samples (0.42%) showing comparatively lower abundance than those from the other two locations, i.e., 3.8% in Kaysville and 2.35% in Campus. *Variibacter* (1.31%) is present in comparatively lower abundance in Kaysville samples than those from Campus (2.77%) and Greenville (3.46%).

In rhizosphere samples, prominent genera include *Blastococcus, Bradyrhizobium, JC017, Novosphingobium, Povalibacter, Pseudomonas, Rhodococcus, Skermanella, Sphingomicrobium*, and *Streptomyces* (Figure 4B). There is no such genus that is significantly higher (*p* < 0.05) than other genera in Greenville rhizosphere samples; however, *Rhodococcus* (7.78%), *JC017* (6.76%), and Streptomyces (4.67%) are strongly present. However, in Kaysville rhizosphere samples, *Pseudomonas* (9.10%) is statistically higher (0.05), and in the Campus rhizosphere samples, the presence of JC017 (12.27%) is significantly higher (*p* < 0.05). While *Rhodococcus* predominates in Greenville (7.78%), its abundance is notably lower in USU Campus (0.17%) samples and absent in Kaysville samples. Conversely, *Pseudomonas*, the most abundant genus in Kaysville (9.10%), exhibits diminished presence in Greenville (0.95%) and USU Campus (2.50%) samples. The genus *Bradyrhizobium* is found in smaller abundance in Greenville (0.37%) compared to other locations i.e. 2.23% in Kaysville and 2.11% in Campus, a trend shared with *Blastococcus, Skermanella, and Streptomyces* which are less abundant in USU Campus samples (0.41%, 0.55%, and 0.19% respectively) than in Greenville (2.79, 1.58, and 4.67% respectively) and Kaysville (1.63%, 2.96%, and 3.48% respectively). *JC017*, which is significantly superior (*p* < 0.05) in terms of abundance in the Campus samples (12.27%), is comparatively lower in the samples from Greenville (6.76%) and Kaysville (3.37%).

*Frankia* is the most prevalent genus in root samples from all three locations, with a corresponding abundance of 58.66, 52.52, and 44.47% in Greenville, Kaysville, and Campus samples, respectively (Figure 4C). The genus *Frankia* was significantly higher (*p* < 0.05) than other genera in the root samples from Kaysville. However, in the case of the root samples from Greenville and Campus, no genus was significantly superior (*p* < 0.05) to the others. Other prominent genera present in the root samples of hybrid buffaloberry are *Bradyrhizobium*, *Streptomyces, Conyzicola, Cutibacterium, Sphingomonas, Actinoplanes, Promicromonospora, Paenibacillus*, *Pseudomonas_E_647464*. *Streptomyces* and Bradyrhizobium, which are present in higher abundance in the Greenville samples (21.63 and 14.05%, respectively), are present in smaller abundance in the samples from Kaysville (9.15 and 3.30%, respectively), and Campus (5.20 and 5.33% respectively). The presence of *Pseudomonas_E_647464* is comparatively higher in the Kaysville samples (11.18%), while its abundance is meager in the Greenville samples (0.39%) and is absent in the samples from the Campus. The genus *Conyzicola*, present in the Campus samples (4.65%), is absent in the samples from Kaysville and Greenville, a trend similar to *Paenibacillus*, which is present in the Kaysville samples (2.87%) and absent in the samples from Greenville and the Campus. *Promicromonospora*, present in both the Greenville samples (1.8%) and the Campus samples (0.42%), is absent in the samples from Kaysville. Moreover, the genera *Actinoplanes, Cutibacterium*, and *Sphingomonas*, which are present in the samples from Kaysville (1.29%, 1.40%, and 0.78%) and Campus (2.09%, 2.82%, and 2.35%) are notably absent in the Greenville samples.

The genus *Frankia* is predominant in nodules, with 99.55% abundance in Kaysville, 94.22% in the Campus, and 91.13% in Greenville (Figure 4D). It is statistically superior (*p* < 0.05) in all three locations compared to other genera. Other genera present in the nodule samples are *Streptomyces, Pseudonocardia, Amycolatopsis_D_380830, Bradyrhizobium, Promicromonospora, Sphingomonas, Sphingobacterium, Pseudomonas_E_647464*, and *Agrobacterium. Streptomyces_G_ 399870* (0.16%) and *Sphingomonas_L_486704* (0.12%) are the only two genera present in the Kaysville samples other than *Frankia* (99.55%). *Agrobacterium* (0.60%) and *Pseudomonas_E_647464* (0.84%) exclusively appear in the samples from Greenville, whereas *Pseudonocardia* (0.58%) and *Amycolatopsis_D_380830* (0.50%) are exclusive to the samples from USU Campus. *Bradyrhizobium* (1.03% in Greenville and 0.3% in Campus), *Promicromonospora* (0.11%in Greenville and 0.20% in Campus), and *Sphingobacterium* (0.63% in Greenville and 0.02% in Campus) are absent in the Kaysville samples. Still, they are in traces in the samples from the other two locations.

We also performed Canonical Correspondence Analysis (CCA) to study the relationship between the bacterial community structure in the soil and root samples of hybrid buffaloberry across the three different locations. The bulk soil and rhizosphere samples from the three locations are separated in the CCA plot (Supplementary Figure 4A), indicating distinct differences in the microbiome composition in the bulk soil and rhizosphere among the locations. However, the root and nodule samples from the three locations are almost clustered in the CCA plot (Supplementary Figure 4B), indicating similar bacterial communities in the root and nodules of hybrid buffaloberry across the different locations. The results of CCA are similar to those obtained from PCoA. The taxonomic classification also suggests that the phylum *Actinobacteriota* is dominant in the root and nodule samples from all three locations, which might be the reason for root and nodule samples clustering in the CCA plot.

### 3.6 Fifty-nine isolates showing different plant growth promoting (PGP) traits were isolated

**FIGURE 5:**
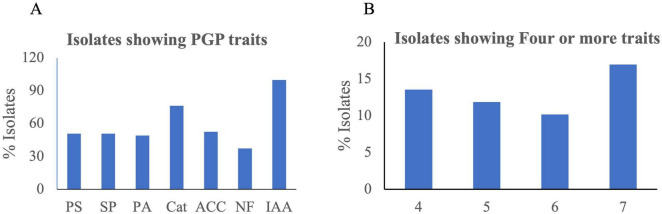
The bar chart **(A)** The percentage of isolates positive for each plant growth promotion trait **(B)** the number of isolates positive for four or more traits.

Fifty-nine bacterial strains (Supplementary Tables 1, 2) were isolated from different media and stored at −80°C as glycerol stocks. Among them, 18 were isolated from 1/4 Nutrient Agar medium, 13 from DF minimal medium with ACC as nitrogen source, nine from Yeast Mannitol Agar medium, eight from Actinomyces Isolation Agar medium, seven from Minimal medium, and four from Potato Dextrose Agar. Of 59 isolates, 25 (42%) were capable of phosphate solubilization, 30 (50%) were siderophore-producing bacteria, 29 (49%) had protease activity, and 45 (76%) showed catalase activity. Similarly, 31 (52%) isolates had ACC deaminase activity, and 22 (37%) were positive for nitrogen fixing capability. All 59 isolates showed IAA production in different concentrations ranging from 1.8 ^± 0.12^ to 23.6 ^± 0.47^ μg/ml (Figure 5A and Supplementary Table 2). Isolates SUWK9 and SUWK19 produced the highest amount of IAA, 23.6 ^± 0.47^ and 23.0 ^± 0.43^ respectively. Thirty-one isolates showed at least four plant growth-promoting traits (Table 3) and 8 of them were able to produce more than 10 μg/ml IAA (Table 4). 14 % (8/59) isolates showed at least four PGP traits, 12% (7/59) showed five PGP traits, 10% (6/59) showed at least six PGP traits and 17% (10/59) showed all seven PGP traits (Figure 5B and Supplementary Tables 2, 3). These ten isolates are SUWK1, SUWK7, SUWK16, SUWK24, SUWK29, SUWK34, SUWK47, SUWK49, SUWK50, and SUWK55 (Table 5). We sequenced nineteen isolates; however, only eleven exhibited homologies with entries in the 16S rRNA database, while the remaining eight did not find a match within the database (Table 3). The identified bacteria belonged to the genera *Stenotrophomonas, Chryseobacterium, Massilia, Variovorax*, and *Pseudomonas*. All of them exhibited four or more PGP traits except SUWK58, which is *Stenotrophomonas* and exhibits only three PGP traits (Supplementary Table 2).

**TABLE 3:** List of Isolates showing four or more PGP traits.

S.No.	Name	PSI	PA	SP	ACC	Cat	IAA (μg/ml)	NF	16S rRNA	Accession No.
1	SUWK1	2.2 ^± 0.11^	++	+	+++	++	4.9 ^± 0.12^	+	ND	−
2	SUWK4	−	+++	+++	++	+	4.6 ^± 0.02^	−	No match	−
3	SUWK5	2.1 ^± 0.10^	++	−	+++	++	7.4 ^± 0.43^	+	*Pseudomonas*	PP430627
4	SUWK6	−	+	+	+++	++	7.3 ^± 0.18^	++	*Massilia*	PP430628
5	SUWK7	2.1 ^± 0.08^	++	++	+++	++	8.9 ^± 0.18^	++	No match	−
6	SUWK9	−	+	+	++	−	23.6 ^± 0.47^	−	*Massilia*	PP430629
7	SUWK11	−	++	−	++	+++	9.0 ^± 0.24^	−	No match	−
8	SUWK12	−	++	−	+++	++	5.4 ^± 0.22^	−	ND	−
9	SUWK16	2.0 ^± 0.06^	++	++	+++	+	4.7 ^± 0.25^	+	*Stenotrophomonas*	PP430630
10	SUWK18	2.0 ^± 0.03^	−	+++	−	+++	3.8 ^± 0.10^	−	ND	−
11	SUWK19	−	+++	+	−	+	23.0 ^± 0.43^	−	ND	−
12	SUWK24	2.0 ^± 0.03^	+	++	++	+	3.1 ^± 0.03^	+	ND	−
13	SUWK25	2.3 ^± 0.10^	−	++	++	+++	4.8 ^± 0.07^	−	No Match	−
14	SUWK29	2.5 ^± 0.12^	++	++	++	+	11.3 ^± 1.33^	++	ND	−
15	SUWK34	2.0 ^± 0.03^	++	+	++	++	15.4 ^± 0.28^	++	No match	−
16	SUWK36	2.2 ^± 0.03^	+	++	−	+	5.0 ^± 0.09^	+++	*Pseudomonas*	PP430631
17	SUWK37	−	+++	+++	−	+	17.2 ^± 1.99^	−	*Chryseobacterium*	PP430632
18	SUWK39	2.2 ^± 0.06^	−	++	−	++	6.6 ^± 0.36^	+	ND	−
19	SUWK40	2.4 ^± 0.05^	−	++	−	++	4.9 ^± 0.09^	−	ND	−
20	SUWK42	2.0 ^± 0.06^	−	++	−	+	8.8 ^± 0.09^	++	ND	−
21	SUWK47	2.3 ^± 0.06^	+++	++	++++	+++	6.7 ^± 0.18^	+++	*Chryseobacterium*	PP430633
22	SUWK48	2.2 ^± 0.08^	−	+++	++	+	6.7 ^± 0.37^	−	ND	−
23	SUWK49	2.0 ^± 0.03^	++	+	+++	+++	7.1 ^± 0.16^	+++	ND	−
24	SUWK50	2.0 ^± 0.03^	++	+	++	+	9.1 ^± 0.25^	++	No match	−
25	SUWK51	−	−	−	++	+	5.2 ^± 0.36^	++	ND	−
26	SUWK52	2.1 ^± 0.05^	+	++	++	+	6.0 ^± 0.19^	−	No Match	−
27	SUWK53	2.3 ^± 0.06^	−	++	++	+	5.5 ^± 0.61^	−	*Stenotrophomonas*	PP430634
28	SUWK54	2.0 ^± 0.03^	−	+	++	++	8.5 ^± 0.43^	++	*Stenotrophomonas*	PP430635
29	SUWK55	2.1 ^± 0.05^	+	++	++	+	5.3 ^± 0.33^	+	ND	−
30	SUWK56	−	+++	+	+++	+	5.2 ^± 0.20^	−	*Variovorax*	PP430636
31	SUWK57	−	++	−	++	+++	8.2 ^± 0.10^	+++	No Match	−

‘−’ negative/ absent, ‘+’ mild positive/ present, ‘++’ moderately positive, ‘+++’ strongly positive, SP, siderophore production; PSI, phosphate solubilization index; IAA, Indole Acetic Acid production (μg/mL); ACC, ACC deaminase activity; PA, protease activity; Cat, catalase production; NF, nitrogen fixation; ND, not done.

**TABLE 4:** List of isolates capable of producing > 10 μg/ml IAA.

S.No	Name	IAA (μg/ml)	16s rRNA
1	SUWK9	23.6 ^± 0.47^	*Massilia*
2	SUWK14	10.6 ^± 0.20^	ND
3	SUWK19	23.0 ^± 0.43^	ND
4	SUWK26	10.3 ^± 0.80^	ND
5	SUWK29	11.3 ^± 1.33^	ND
6	SUWK34	15.4 ^± 0.28^	No Match
7	SUWK37	17.2 ^± 1.99^	*Chryseobacterium*
8	SUWK41	12.8 ^± 0.31^	ND

ND, not done.

**TABLE 5:** Isolates positive for all seven tested plant growth promotion traits.

S.No.	Name	PSI	PA	SP	ACC	Cat	IAA (μg/ml)	NF	16S rRNA	Accession No.
1	SUWK1	2.2 ^± 0.11^	++	+	+++	++	5.4	+	ND	−
2	SUWK7	2.1 ^± 0.08^	++	++	+++	++	9.4	++	No match	−
3	SUWK16	2.0 ^± 0.06^	++	++	+++	+	5.3	+	*Stenotrophomonas*	PP430630
4	SUWK24	2.0 ^± 0.03^	+	++	++	+	3.7	+	ND	−
5	SUWK29	2.5 ^± 0.12^	++	++	++	+	11.8	++	ND	−
6	SUWK34	2.0 ^± 0.03^	++	+	++	++	15.9	++	No match	−
7	SUWK47	2.3 ^± 0.06^	+++	++	++++	+++	7.2	+++	*Chryseobacterium*	PP430633
8	SUWK49	2.0 ^± 0.03^	++	+	+++	+++	7.6	+++	ND	−
9	SUWK50	2.0 ^± 0.03^	++	+	++	+	9.7	++	No match	−
10	SUWK55	2.1 ^± 0.05^	+	++	++	+	5.8	+	ND	−

‘−’ negative/absent, ‘+’ mild positive/present, ‘++’ moderately positive, ‘+++’ strongly positive, SP, siderophore production; PSI, phosphate solubilization index; IAA, Indole Acetic Acid production (μg/mL); ACC, ACC deaminase activity; PA, protease activity; Cat, catalase production; NF, nitrogen fixation; ND, not done.

## 4 Discussion

Plant microbiome plays a significant role in plant’s growth and development. Similarly, geography and management also affect the soil microbiome (Coller et al., 2019). Long-term land use significantly affects soil microbial communities (Goss-Souza et al., 2017). Our study found that the alpha diversity, i.e., the richness and evenness of the bulk soil, rhizosphere, and root bacteria, are similar in all three locations. Utah State University manages and takes care of all three locations well. They are well fertilized and well manured so that the soil is rich in organic matter and nutrient elements, which makes them suitable habitats for large numbers of bacteria.

Moreover, the soil type is also similar among the three locations, i.e., loam, which might be the reason for the similarity in the number and evenness of bacteria in the soil and root. Using external organic material in soil increased functional and genetic microbial diversity (Gryta et al., 2020). There was a significant increase in microbial diversity and community composition with the addition of pig manure and crop residue in the soil in different experiments (Li et al., 2020; Lundquist et al., 1999). Adding pig manure and rice straw to soil increased the microbial diversity and community, increasing the ratio of gram-positive to gram-negative bacteria (Liu et al., 2023). (Liu et al., 2023). However, the Chao1 index (i.e., richness) of bacteria in nodules is significantly lower in Kaysville samples than those from Greenville and USU logan Campus samples; this might be due to the high salt concentration in the bulk soil of Kaysville (Figure 1D, Table 2, and Supplementary Tables 12, 13). Numerous reports highlight the interaction of microbial endophytes in enhancing tolerance to both biotic and abiotic stresses in plants (Kuldau and Bacon, 2008; Waller et al., 2005).

The principal Co-ordinate Analysis (PCoA) and PERMANOVA test, the robust statistical methods, were used to analyze the bacterial community based on the Bray-Curtis dissimilarity matrix. These tests revealed significant differences (*p* < 0.05) in bulk soil and nodule bacterial composition among the three locations (Figures 2A, D and Supplementary Tables 5, 15). However, there was no significant difference (*p* < 0.05) in the rhizosphere and root bacterial composition among the three locations (Figures 2B, C and Supplementary Tables 8, 11). The composition of the rhizosphere microbiome is influenced by root exudates, plant genotype, and prevailing environmental factors (Kawasaki et al., 2016). Significant alterations in the composition of the root microbial community were reported, including changes in the composition of root exudates due to varying iron nutritional status (Yang and Crowley, 2000). A research investigation on rice, conducted in greenhouse and field conditions, revealed distinct patterns in the root microbiome composition. In the greenhouse setting, variations were observed based on genotype and soil source. Conversely, under field conditions, the root microbiome composition exhibited differences correlated with geographical location and cultivation practices, specifically whether the cultivation was organic or conventional (Edwards et al., 2015). The difference in the bulk soil and rhizosphere microbial community of *Cinmaomum migao*, a rare and endangered species exclusive in China, with changes in time and location, was reported (Chen et al., 2023).

*Proteobacteria, Actinobacteriota*, and *Acidobacteriota* emerge as the predominant phyla in bulk soil samples across all surveyed locations, grossing almost 72% of the total phyla; however, in different proportions (Figure 3A). This diversity in proportions across locations highlights the complexity of the microbial world. Regarding the bacterial community composition in the rhizosphere samples of hybrid buffaloberry from the three locations, *Proteobacteria, Actinobacteriota, Bacteroidota*, and *Acidobacteriota* are the top phyla contributing almost 89% in Greenville and nearly 85% in Kaysville and the Campus samples with different composition in each location (Figure 3B). The bacterial communities in terms of the genus in the bulk soil and rhizosphere are diverse (Figures 4A, B); however, *Frankia* is the dominant genus in the root and root nodule samples, although in different level of abundance (Figures 4C, D). Though *Frankia* dominates in the root and nodule samples of hybrid buffaloberry, the presence and dominance of other genera greatly vary among different locations. Similarly, a study reported that *Actinobacteria* was the dominant phylum with 97.73% relative abundance in the bulk soil samples of a Moroccan phosphate mine, while *Proteobacteria* (62.24%, 71.15% and 65.61%), *Actinobacteria* (22.53%, 15.24%, 22.30%), *Bacteroidetes* (7.57%; 4.23%; 7.63%), and *Firmicutes* (5.82%; 1.17%; 2.83%) were the dominant phyla in the rhizosphere of three native plants viz *Alternanthera caracasana*, *Digitaria sanguinalis*, and *Dittrichia Viscosa* in the same study. Another study on Poplar in Tennessee and North Carolina, identified variations in the rhizosphere and root microbiome (Shakya et al., 2013). Bulk soil samples from forests in China were collected and analyzed for microbial community composition and structure, which identified *Bradyrhizobium* and *Methylobacterium* as the major genera in the samples (Meng et al., 2019).

Metagenomic study of the rhizosphere of the three native *plants* in Kuwait revealed Rhizobium as the predominant genus in *Rhanterium epapposum and Haloxylon salicornicum;* however, *Pseudomonas* was the predominant genus in *Farsetia aegyptia.* Additionally, the study investigated the root nodule microbiome of another native plant, *Vachellia pachyceras*, identifying *Agrobacterium* as the dominant genus, alongside the notable presence of *Cellulomonas, Bacillus, and Pseudomonas* (Suleiman et al., 2019). In the case of actinorhizal plants *Alnus* spp. (alder), *Frankia* was the dominant genus in the root nodules of *Alnus formosana* (24.54%) and *Alnus glutinosa* (56.90%), whereas *Pseudonocardia* (26.21%) dominated in *Alnus cremastogyne* (Yuan et al., 2023). Root nodule microbiome study of *Falcataria falcata*, an exotic tree species in southern China, revealed *Bradyrhizobium* (13–37%) and *Paucibacter* (1–34%) as the dominant genera; however, *Bradyrhizobium, Paucibacter, Rhizobium*, and *Mesorhizobium* were consistently present in almost all the samples (Xiang et al., 2023).

A diverse group of Plant growth-promoting bacteria belonging to *Pseudomonas*, *Chryseobacterium*, *Stenotrophomonas*, *Massilia*, *Serratia*, and *Variovorax* was isolated from our study (Table 3 and Supplementary Table 2). Our results show that all our sequenced bacteria except SUWK58 have at least four plant growth-promoting (PGP) traits (Supplementary Table 2). The isolate SUWK16, identified as *Stenotrophomonas* isolates, showed all seven tested PGP traits. The two isolates, SUWK53 and SUWK54, identified as *Stenotrophomonas* (Table 3), are positive for five and six PGP traits respectively. *Stenotrophomonas* available in different environmental conditions have strong potential to be used as Plant growth-promoting bacteria; they can form a biofilm which helps them colonize effectively in the host plant along with phosphate solubilization, siderophore production, IAA production, and Cytokinin production activity (Zhao et al., 2024).

Two isolates, SUWK5 and SUWK36, which are *Pseudomonas*, were positive for six PGP traits. SUWK5 did not have protease activity, and SUWK 36 could not grow on ACC (Table 3). There are many reports of beneficial activities of plant-associated *Pseudomonas*, like suppression of Plant pathogenic microorganisms, synthesis of growth-stimulating phytohormones, and promotion of plant disease resistance (Preston, 2004; Sah et al., 2021). Moreover, they are also involved in the production of siderophores, hydrolytic enzymes like β-1,3-glucanase and chitinases, and other metabolites like phytoalexins (David et al., 2018). PGPR isolated from *C. velutinus* rhizosphere belonged to *Pseudomonas* and exhibited several plant growth-promoting activities like IAA production, Phosphate solubilization, siderophore and catalase production, protease activity, and ACC deaminase activities (Ganesh et al., 2022, 2024).

SUWK6 and SUWK9 sequenced as *Massilia* species, showed six and four PGP traits, respectively (Table 3). *Massilia* species promote the colonization of plant roots by some beneficial microbes like arbuscular mycorrhizal fungi (Peta et al., 2019) and also help in the solubilization of recalcitrant phosphate in soil, which promotes phosphate availability to plants (Zheng et al., 2017). The isolate SUWK56 was identified as *Variovorax* and exhibited five plant growth-promoting (PGP) traits. There are reports that the rhizobacteria *Variovorax* has beneficial plant-microbe interactions (Sun et al., 2018). Likewise, among the two sequenced *Chryseobacterium* isolates, SUWK47 was positive for all seven PGP traits, while SUWK37 showed four PGP traits. *Chryseobacterium* is reported to have beneficial characteristics involved in biocontrol and plant growth promotion (Jung et al., 2023). Out of nineteen sequenced bacteria, eight were unidentified. They did not match with the 16S rRNA database, which could be novel Plant growth-promoting Rhizobacteria (PGPR) as they possess at least four plant growth-promoting (PGP) traits.

## 5 Conclusion

The hybrid buffaloberry is a relatively new interspecific hybrid that has been developed, and our research is shedding light on its microbiological aspects. Despite the variation in location, the microbial ecosystem seems stable as there is consistent alpha diversity in bulk soil, rhizosphere, and root among the studied environments. All the plant samples collected from well-taken care and maintained farms might be the reason for a stable ecosystem in different locations. The influence of location-specific factors on shaping the microbial population is particularly noticed in bulk soil and nodule samples, which might be attributed to differences in soil properties or other environmental variables. The dominance of *Proteobacteria* and *Actinobacteriota* in bulk soil and rhizosphere highlights their importance in plant-microbe interactions; bacteria belonging to these phyla might be the key players in plant-microbe interaction. The sole dominance of *Actinobacteriota* in the root and nodule samples indicates their significance in root-associated functions as endophytes. Though there was considerable genus diversity in the bulk soil and rhizosphere, the dominance of *Frankia* in the root and nodule samples suggests a symbiotic relationship between *S. utahensis* and *Frankia*, potentially contributing to plant growth and resilience. Thirty-one bacterial isolates showed at least four plant growth-promoting traits, indicating microbial communities’ role in promoting plant health and resilience in adverse environments. This study obtained a list of 10 plant growth-promoting bacteria that exhibit all seven PGP traits tested in this study. Climate-resilient landscaping strategies could be developed efficiently by studying and understanding the dynamics of soil and root microbiomes associated with native plants like *S. utahensis.* The beneficial properties of the microbial communities associated with native hardy plant species can be harnessed to aid in mitigating the impacts of climate change, enhancing ecosystem resilience, preserving biodiversity, developing biofertilizers/ biostimulants, and sustainable agriculture. These potential applications of our research inspire us to continue our work in this field.

## Data Availability

The 16S rRNA sequence data generated in the study are deposited in the GenBank NCBI repository, accession numbers PP430627, PP430628, PP430629, PP430630, PP430631, PP430632, PP430633, PP430634, PP430635, PP430636, and PP430637. The metagenomic data generated during the study are deposited SRA NCBI repository Bio project PRJNA1084074.

## Footnotes

1
https://en.novogene.com/16s-18s-its-amplicon-metagenomic-sequencing/#overview
