# Supplementary material for: Soil and root microbiome analysis and isolation of plant growth-promoting bacteria from hybrid buffaloberry (Shepherdia utahensis ‘Torrey’) across three locations
Source: Front Microbiol. 2024 Sep 9;15:1396064. doi: 10.3389/fmicb.2024.1396064 (PMC11417967; doi:10.3389/fmicb.2024.1396064)
Supplement: Supplementary file 1 [file Data_Sheet_1.docx]

Supplementary Material

# Supplementary Data

The 16S rRNA sequences are submitted to GenBank NCBI with submission no. SUB14284866 and accession numbers are given in the table 3 and S2. The metagenomic data is submitted to SRA Bio project [PRJNA1084074.](https://www.ncbi.nlm.nih.gov/bioproject/1084074)

# Supplementary Figures and Tables

**Table S1 . Morphological characteristics of all the isolates from the rhizosphere of hybrid buffaloberry**

| **Name** | **Size** | **Shape** | **Margin** | **Color** | **Opacity** | **Elevation** | **Surface** | **16S rRNA** | **Accession No.** |
| --- | --- | --- | --- | --- | --- | --- | --- | --- | --- |
| SUWK1 | medium | Round | Entire | Clear/  off-white | TT | Flat | Smooth | ND | - |
| SUWK2 | medium | Irregular | Undulated | Yellow | TP | Flat | Glistening | ND | - |
| SUWK3 | large | Round | Entire | Off-white | TP | Raised | Smooth | ND | - |
| SUWK4 | large/ spreading | Irregular | Lobate | Yellow | TP | Raised | Smooth | No match | - |
| SUWK5 | small | Round | Entire | White | OP | Raised | Smooth | *Pseudomonas* | PP430627 |
| SUWK6 | small | Round | Lobate | Clear | TP | Flat | Smooth | *Massilia* | PP430628 |
| SUWK7 | large/  spreading | Irregular | Lobate | White | OP | Flat | Smooth | No match |  |
| SUWK8 | small | Irregular | Lobate | White | OP | Flat | Dull | ND | - |
| SUWK9 | small | Round | Entire | Yellow | TP | Flat | Glistening | *Massilia* | PP430629 |
| SUWK10 | small | Irregular | Undulated | Off-white | TT | Raised | Smooth | ND | - |
| SUWK11 | medium | Round | Lobate | Clear | TP | Flat | Smooth | No match | - |
| SUWK12 | small | Round | Entire | Off-white | OP | Flat | Dull | ND | - |
| SUWK13 | small | Irregular | Lobate | Clear | TP | Raised | Smooth | ND | - |
| SUWK14 | small | Irregular | Puniform | Yellow | OP | Flat | Dull | ND | - |
| SUWK15 | medium | Irregular | Lobate | White | TT | Flat | Smooth | ND | - |
| SUWK16 | small | Irregular | Undulated | Yellow | TP | Flat | Glistening | *Stenotrophomonas* | PP430630 |
| SUWK17 | medium | Irregular | Lobate | Off-white | OP | Raised | Smooth | ND | - |
| SUWK18 | small | Irregular | Puniform | Yellow | TT | Flat | Dull | ND | - |
| SUWK19 | small | Round | Entire | White | OP | Raised | Smooth | ND | - |
| SUWK20 | small | Irregular | Lobate | Clear/  White | TT | Raised | Smooth | ND | - |
| SUWK21 | punctiform | Irregular | Lobate | Clear/  White | TT | Flat | Smooth | ND | - |
| SUWK22 | small | Irregular | Undulated | Yellow | OP | Flat | Dull | ND | - |
| SUWK23 | small | Irregular | Lobate | Yellow | OP | Flat | Glistening | ND | - |
| SUWK24 | small | Irregular | Lobate | Off-white | OP | Raised | Smooth | ND | - |
| SUWK25 | small | Round | Entire | White | TT | Raised | Smooth | No match | - |
| SUWK26 | punctiform | circular | entire | white/  off-white | OP | pulvinate | dull | ND | - |
| SUWK27 | punctiform | circular | entire | white/  off-white | OP | pulvinate | dull | ND | - |
| SUWK28 | medium | circular | undulate | grey | OP | flat | dull | ND | - |
| SUWK29 | medium | irregular | undulate | White/yellow | OP | convex | dull | ND | - |
| SUWK30 | small | circular | entire | mustard yellow | TT | convex | smooth | ND | - |
| SUWK31 | small | circular | entire | mustard yellow | TT | convex | smooth | ND | - |
| SUWK32 | medium | circular | entire | white/  off-white | OP | umbonate | dull | ND | - |
| SUWK33 | medium | circular | entire | Off-white | OP | convex | dull | ND | - |
| SUWK34 | medium | circular | entire | white | OP | convex | smooth | No match | - |
| SUWK35 | punctiform | circular | entire | yellow | TT | convex | smooth | ND | - |
| SUWK36 | large/  spreading | irregular | lobate | Off-white/  clear | TP | flat | dull | *Pseudomonas* | PP430631 |
| SUWK37 | small | irregular | entire | vibrant yellow | TT | convex | smooth | *Chryseobacterium* | PP430632 |
| SUWK38 | large | filamentous | curled | clear | TP | flat/raised | dull | ND | - |
| SUWK39 | punctiform | circular | entire | yellow | TT | convex | smooth | ND | - |
| SUWK40 | punctiform | circular | rhizoid | offwhite/  brownish | TP | flat | dull | ND | - |
| SUWK41 | small | circular | entire | white/tan | OP | flat/  umbonate | dull | ND | - |
| SUWK42 | punctiform | circular | entire | white | OP | pulvinate | dull | ND | - |
| SUWK43 | punctiform | circular | entire | white/  off-white | OP | flat | dull | ND | - |
| SUWK44 | large/  spreading | circular | entire | yellow | TT | pulvinate | smooth | ND | - |
| SUWK45 | medium/  spreading | irregular | lobate | yellow/  clear | TP | flat | smooth | ND | - |
| SUWK46 | small | circular | entire | clear | TT | convex | smooth | ND | - |
| SUWK47 | large/  spreading | irregular | lobate | yellow | TP | flat | smooth | *Chryseobacterium* | PP430633 |
| SUWK48 | medium | irregular | lobate | clear | TP | flat | smooth | ND | - |
| SUWK49 | medium | irregular | undulate | white/clear | OP | convex | dull | ND | - |
| SUWK50 | medium | circular | entire | yellow/  off-white | TP | convex | smooth | No match | - |
| SUWK51 | medium | circular | entire | off-white | TP | flat | smooth | ND | - |
| SUWK52 | small | irregular | lobate | clear | TT | convex | smooth | No match | - |
| SUWK53 | large | irregular | lobate | yellow/off-white | TP | flat | smooth | *Stenotrophomonas* | PP430634 |
| SUWK54 | medium | irregular | undulate | clear/off-white | TT | raised | smooth | *Stenotrophomonas* | PP430635 |
| SUWK55 | medium | circular | entire | yellow | TP | convex | smooth | ND | - |
| SUWK56 | large/ spreading | irregular | lobate | yellow/off-white | TP | flat | smooth | *Variovorax* | PP430636 |
| SUWK57 | medium | irregular | puniform | clear | TT | flat | glistening | No match | - |
| SUWK58 | large | circular | entire | clear/off-white | TP | flat | smooth | *Stenotrophomonas* | PP430637 |
| SUWK59 | medium | irregular | lobate | white/off-white | TP | raised | dull | ND | - |

**Table S2: List of total bacterial isolates from the rhizosphere of hybrid buffaloberry with PGP traits, taxonomy, and Accession number**

| **Name** | **PS** | **PA** | **SP** | **ACC** | **Catalase** | **IAA (mg/ml)** | **NF** | **16S rRNA** | **Accession No.** |
| --- | --- | --- | --- | --- | --- | --- | --- | --- | --- |
| SUWK1 | 2.2^±0.11^ | ++ | + | +++ | ++ | 4.9^±0.12^ | + | ND | - |
| SUWK2 | - | - | - | +++ | ++ | 1.8^±0.12^ | - | ND | - |
| SUWK3 | - | ++ | - | - | +++ | 5.5^±0.16^ | - | ND | - |
| SUWK4 | - | +++ | +++ | ++ | + | 4.6^±0.02^ | - | No match | - |
| SUWK5 | 2.1^±0.10^ | ++ | - | +++ | ++ | 7.4^±0.43^ | + | *Pseudomonas* | PP430627 |
| SUWK6 | - | + | + | +++ | ++ | 7.3^±0.18^ | ++ | *Massilia* | PP430628 |
| SUWK7 | 2.1^±0.08^ | ++ | ++ | +++ | ++ | 8.9^±0.18^ | ++ | No match | - |
| SUWK8 | - | + | - | - | - | 3.9^±0.01^ | - | ND | - |
| SUWK9 | - | + | + | ++ | - | 23.6^±0.47^ | - | *Massilia* | PP430629 |
| SUWK10 | - | - | - | ++ | +++ | 2.6^±0.03^ | - | ND | - |
| SUWK11 | - | ++ | - | ++ | +++ | 9.0^±0.24^ | - | No match | - |
| SUWK12 | - | ++ | - | +++ | ++ | 5.4^±0.22^ | - | ND | - |
| SUWK13 | - | - | + | - | +++ | 7.9^±0.16^ | - | ND | - |
| SUWK14 | - | - | - | - | ++ | 10.6^±0.20^ | + | ND | - |
| SUWK15 | - | ++ | - | - | ++ | 5.6^±0.08^ | - | ND | - |
| SUWK16 | 2.0^±0.06^ | ++ | ++ | +++ | + | 4.7^±0.25^ | + | *Stenotrophomonas* | PP430630 |
| SUWK17 | - | ++ | - | - | +++ | 6.5^±0.20^ | - | ND | - |
| SUWK18 | 2.0^±0.03^ | - | +++ | - | +++ | 3.8^±0.10^ | - | ND | - |
| SUWK19 | - | +++ | + | - | + | 23.0^±0.43^ | - | ND | - |
| SUWK20 | - | +++ | - | - | ++ | 5.0^±0.15^ | - | ND | - |
| SUWK21 | - | - | - | +++ | ++ | 3.6^±0.19^ | + | ND | - |
| SUWK22 | - | - | - | - | + | 4.8^±0.12^ | - | ND | - |
| SUWK23 | - | - | - | - | +++ | 4.4^±0.12^ | - | ND | - |
| SUWK24 | 2.0^±0.03^ | + | ++ | ++ | + | 3.1^±0.03^ | + | ND | - |
| SUWK25 | 2.3^±0.10^ | - | ++ | ++ | +++ | 4.8^±0.07^ | - | No match | - |
| SUWK26 | - | - | - | - | - | 10.3^±0.80^ | - | ND | - |
| SUWK27 | - | - | - | - | - | 4.7^±0.08^ | - | ND | - |
| SUWK28 | - | - | - | - | - | 2.9^±0.04^ | - | ND | - |
| SUWK29 | 2.5^±0.12^ | ++ | ++ | ++ | + | 11.3^±1.33^ | ++ | ND | - |
| SUWK30 | - | - | - | - | - | 5.1^±0.20^ | - | ND | - |
| SUWK31 | - | - | +++ | - | ++ | 9.4^±0.17^ | - | ND | - |
| SUWK32 | 2.0^±0.03^ | - | - | - | - | 1.8^±0.05^ | - | ND | - |
| SUWK33 | 2.0^±0.06^ | - | - | - | - | 2.8^±0.12^ | - | ND | - |
| SUWK34 | 2.0^±0.03^ | ++ | + | ++ | ++ | 15.4^±0.28^ | ++ | No match | - |
| SUWK35 | - | - | - | - | - | 5.7^±0.26^ | - | ND | - |
| SUWK36 | 2.2^±0.03^ | + | ++ | - | + | 5.0^±0.09^ | +++ | *Pseudomonas* | PP430631 |
| SUWK37 | - | +++ | +++ | - | + | 17.2^±1.99^ | - | *Chryseobacterium* | PP430632 |
| SUWK38 | - | + | - | ++ | - | 7.3^±0.24^ | - | ND | - |
| SUWK39 | 2.2^±0.06^ | - | ++ | - | ++ | 6.6^±0.36^ | + | ND | - |
| SUWK40 | 2.4^±0.05^ | - | ++ | - | ++ | 4.9^±0.09^ | - | ND | - |
| SUWK41 | 2.8^±0.05^ | - | - | - | - | 12.8^±0.31^ | + | ND | - |
| SUWK42 | 2.0^±0.06^ | - | ++ | - | + | 8.8^±0.09^ | ++ | ND | - |
| SUWK43 | - | - | - | +++ | ++ | 5.6^±0.06^ | - | ND | - |
| SUWK44 | - | - | - | - | - | 3.3^±0.15^ | + | ND | - |
| SUWK45 | - | - | - | ++ | - | 4.1^±0.15^ | - | ND | - |
| SUWK46 | 2.1^±0.05^ | - | +++ | - | + | 5.7^±0.34^ | - | ND | - |
| SUWK47 | 2.3^±0.06^ | +++ | ++ | ++++ | +++ | 6.7^±0.18^ | +++ | *Chryseobacterium* | PP430633 |
| SUWK48 | 2.2^±0.08^ | - | +++ | ++ | + | 6.7^±0.37^ | - | ND | - |
| SUWK49 | 2.0^±0.03^ | ++ | + | +++ | +++ | 7.1^±0.16^ | +++ | ND | - |
| SUWK50 | 2.0^±0.03^ | ++ | + | ++ | + | 9.1^±0.25^ | ++ | No match | - |
| SUWK51 | - | - | - | ++ | + | 5.2^±0.36^ | ++ | ND | - |
| SUWK52 | 2.1^±0.05^ | + | ++ | ++ | + | 6.0^±0.19^ | - | No match | - |
| SUWK53 | 2.3^±0.06^ | - | ++ | ++ | + | 5.5^±0.61^ | - | *Stenotrophomonas* | PP430634 |
| SUWK54 | 2.0^±0.03^ | - | + | ++ | ++ | 8.5^±0.43^ | ++ | *Stenotrophomonas* | PP430635 |
| SUWK55 | 2.1^±0.05^ | + | ++ | ++ | + | 5.3^±0.33^ | + | ND | - |
| SUWK56 | - | +++ | + | +++ | + | 5.2^±0.20^ | - | *Variovorax* | PP430636 |
| SUWK57 | - | ++ | - | ++ | +++ | 8.2^±0.10^ | +++ | No match | - |
| SUWK58 | - | ++ | - | - | ++ | 5.8^±0.22^ | - | *Stenotrophomonas* | PP430637 |
| SUWK59 | - | - | + | +++ | - | 6.9^±0.56^ | - | ND | - |

**Table S3. ANOVA of Chao1 index of bulk soil microbiome of hybrid buffaloberry from the three locations**

|  | Df | Sum Sq | Mean Sq | F value | Pr(>F) |
| --- | --- | --- | --- | --- | --- |
| Location | 2 | 26.72222 | 13.36111 | 0.029132 | 0.971425 |
| Residuals | 6 | 2751.833 | 458.6389 |  |  |

**Table S4. ANOVA of Shannon index of bulk soil microbiome of hybrid buffaloberry from the three locations**

|  | Df | Sum Sq | Mean Sq | F value | Pr(>F) |
| --- | --- | --- | --- | --- | --- |
| Location | 2 | 0.001057 | 0.000529 | 0.00973 | 0.990332 |
| Residuals | 6 | 0.32601 | 0.054335 |  |  |

**Table S5. PERMANOVA of Bray-Curtis distance of bulk soil microbiome of hybrid buffaloberry from the three locations**

|  | Df | Sum of Sqs | R2 | F | Pr(>F) |
| --- | --- | --- | --- | --- | --- |
| Location | 2 | 0.606831 | 0.378535 | 1.8273 | 0.003 |
| Residual | 6 | 0.996275 | 0.621465 |  |  |
| Total | 8 | 1.603106 | 1 |  |  |

**Table S6. ANOVA of Chao1 index of rhizosphere microbiome of hybrid buffaloberry from the three locations**

|  | Df | Sum Sq | Mean Sq | F value | Pr(>F) |
| --- | --- | --- | --- | --- | --- |
| Location | 2 | 748.0817 | 374.0408 | 0.199057 | 0.824704 |
| Residuals | 6 | 11274.41 | 1879.068 |  |  |

**Table S7. ANOVA of Shannon index of rhizosphere microbiome of hybrid buffaloberry from the three locations**

|  | Df | Sum Sq | Mean Sq | F value | Pr(>F) |
| --- | --- | --- | --- | --- | --- |
| Location | 2 | 0.372482 | 0.186241 | 1.890259 | 0.23087 |
| Residuals | 6 | 0.59116 | 0.098527 |  |  |

**Table S8. PERMANOVA of Bray-Curtis distance of rhizosphere microbiome of hybrid buffaloberry from the three locations**

|  | Df | Sum of Sqs | R2 | F | Pr(>F) |
| --- | --- | --- | --- | --- | --- |
| location | 2 | 0.500794 | 0.293568 | 1.246694 | 0.131 |
| Residual | 6 | 1.205093 | 0.706432 |  |  |
| Total | 8 | 1.705887 | 1 |  |  |

**Table S9. ANOVA of Chao1 index of root endosphere of hybrid buffaloberry from the three locations**

|  | Df | Sum Sq | Mean Sq | F value | Pr(>F) |
| --- | --- | --- | --- | --- | --- |
| Location | 2 | 374.1667 | 187.0833 | 1.666419 | 0.278896 |
| Residuals | 5 | 561.3333 | 112.2667 |  |  |

**Table S10. ANOVA of Shannon index of root endosphere of hybrid buffaloberry from the three locations**

|  | Df | Sum Sq | Mean Sq | F value | Pr(>F) |
| --- | --- | --- | --- | --- | --- |
| Location | 2 | 2.82427 | 1.412135 | 1.440593 | 0.320587 |
| Residuals | 5 | 4.901227 | 0.980245 |  |  |

**Table S11. PERMANOVA of Bray-Curtis distance of root endosphere of hybrid buffaloberry from the three locations**

|  | Df | Sum of Sqs | R2 | F | Pr(>F) |
| --- | --- | --- | --- | --- | --- |
| Location | 2 | 0.517415 | 0.276667 | 0.956223 | 0.567 |
| Residual | 5 | 1.352757 | 0.723333 |  |  |
| Total | 7 | 1.870172 | 1 |  |  |

**Table S12. ANOVA of Chao1 index of nodule endosphere of hybrid buffaloberry from the three locations**

|  | Df | Sum Sq | Mean Sq | F value | Pr(>F) |
| --- | --- | --- | --- | --- | --- |
| Location | 2 | 215.3889 | 107.6944 | 18.20188 | 0.002833 |
| Residuals | 6 | 35.5 | 5.916667 |  |  |

**Table 13. Tukey HSD mean comparison of Chao1 index of nodule endosphere of hybrid buffaloberry from the three locations**

|  | Diff | lwr | upr | p adj |
| --- | --- | --- | --- | --- |
| Greenville-Campus | -2.83333 | -8.92712 | 3.260452 | 0.386772 |
| Kaysville-Campus | -11.5 | -17.5938 | -5.40622 | 0.002809 |
| Kaysville-Greenville | -8.66667 | -14.7605 | -2.57288 | 0.011264 |

**Table S14. ANOVA of Shannon index of nodule endosphere of hybrid buffaloberry from the three locations**

|  | Df | Sum Sq | Mean Sq | F value | Pr(>F) |
| --- | --- | --- | --- | --- | --- |
| Location | 2 | 0.082983 | 0.041492 | 0.547219 | 0.604922 |
| Residuals | 6 | 0.454935 | 0.075823 |  |  |

**Table S15. PERMANOVA of Bray-Curtis distance of nodule endosphere of hybrid buffaloberry from the three locations**

|  | Df | Sum of Sqs | R2 | F | Pr(>F) |
| --- | --- | --- | --- | --- | --- |
| Location | 2 | 0.83536 | 0.465238 | 2.609975 | 0.006 |
| Residual | 6 | 0.960193 | 0.534762 |  |  |
| Total | 8 | 1.795552 | 1 |  |  |

Figure S1


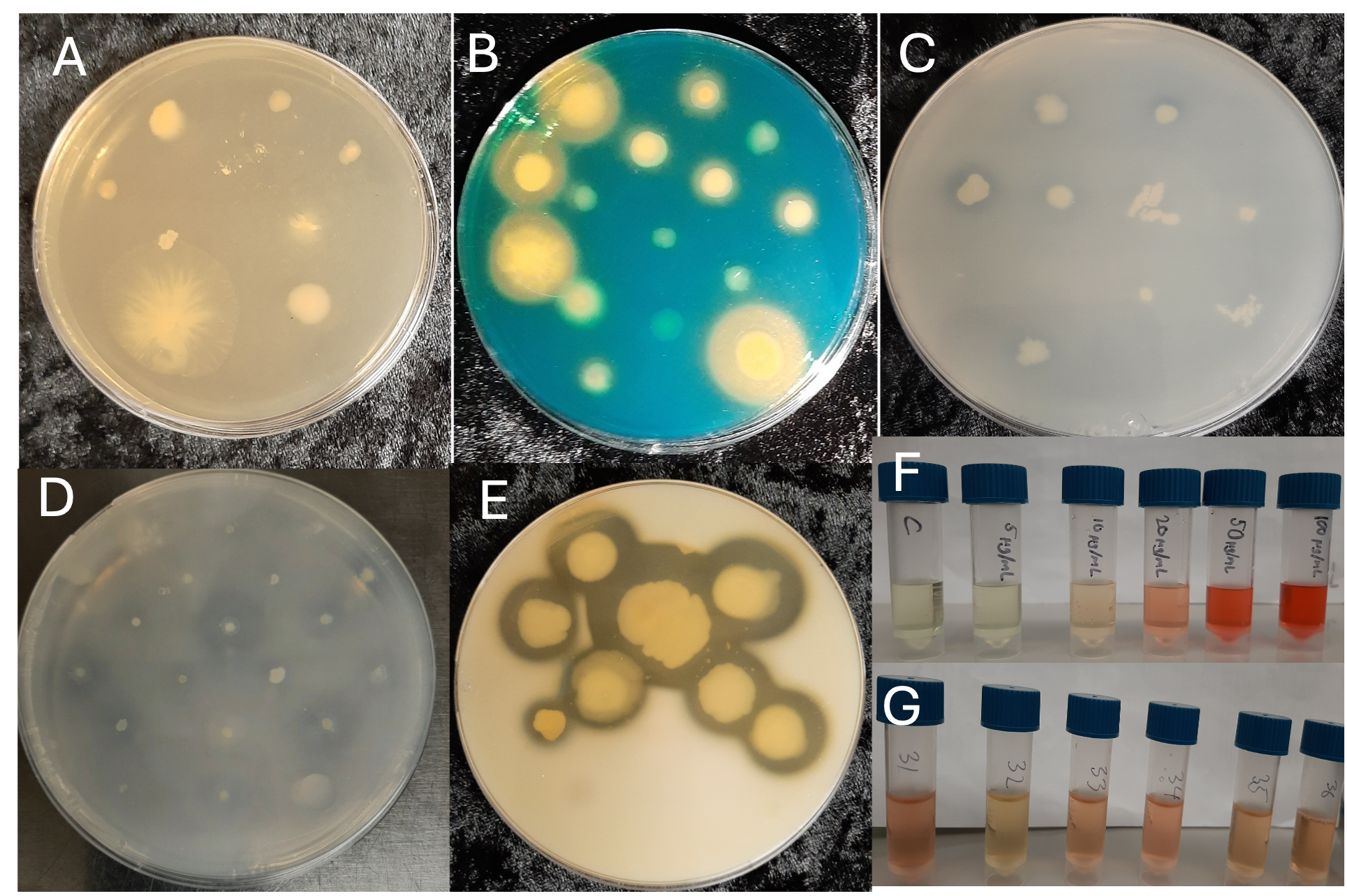


Figure S1. Representative isolates for plant growth promoting traits on A) DF-ACC media B) CAS media C) Pikovskaya Media D) Norris Glucose Nitrogen Free Media E) Skim milk Agar F) IAA assay standard Curve G) IAA assay

Figure S2


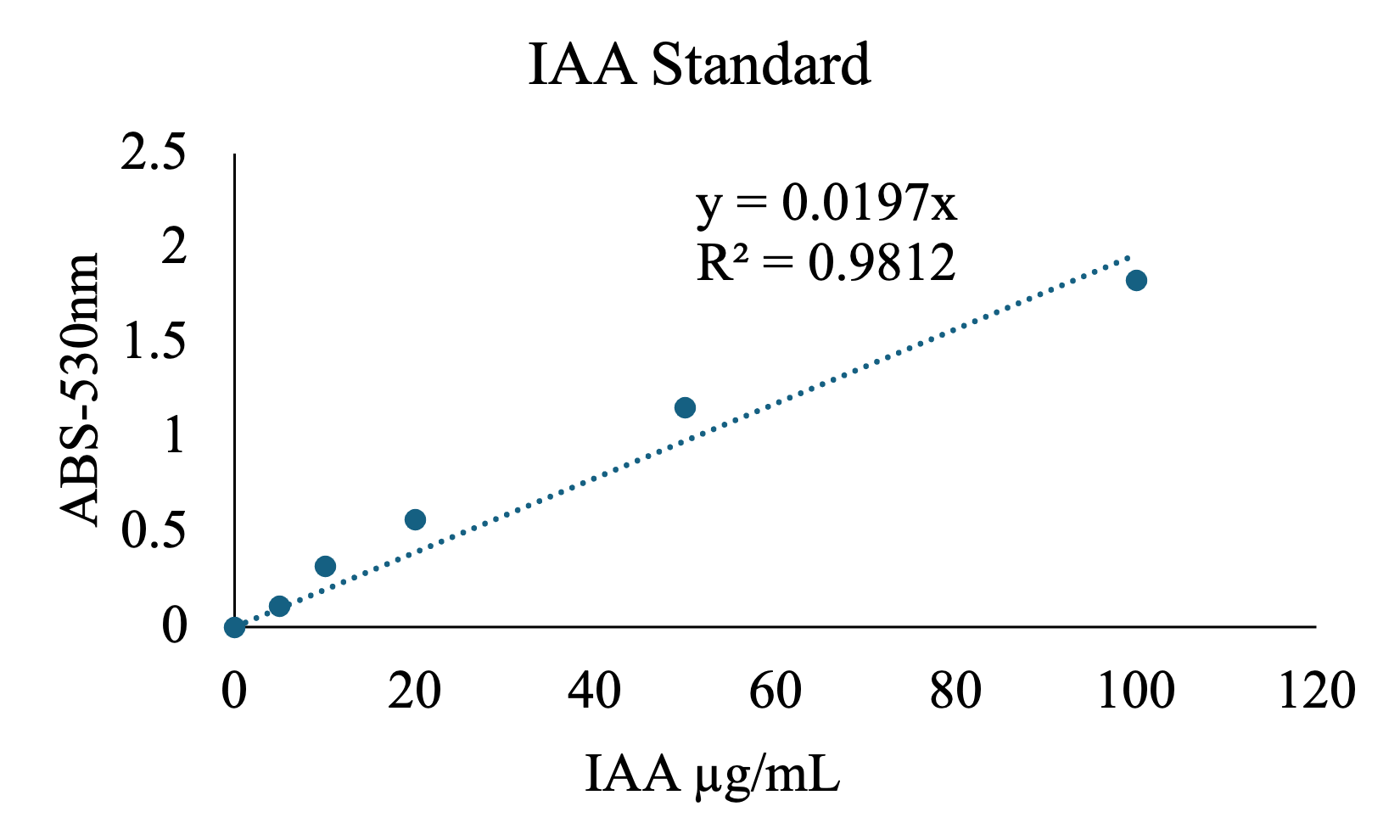


Figure S2 IAA standard curve.

Figure S3 Histogram


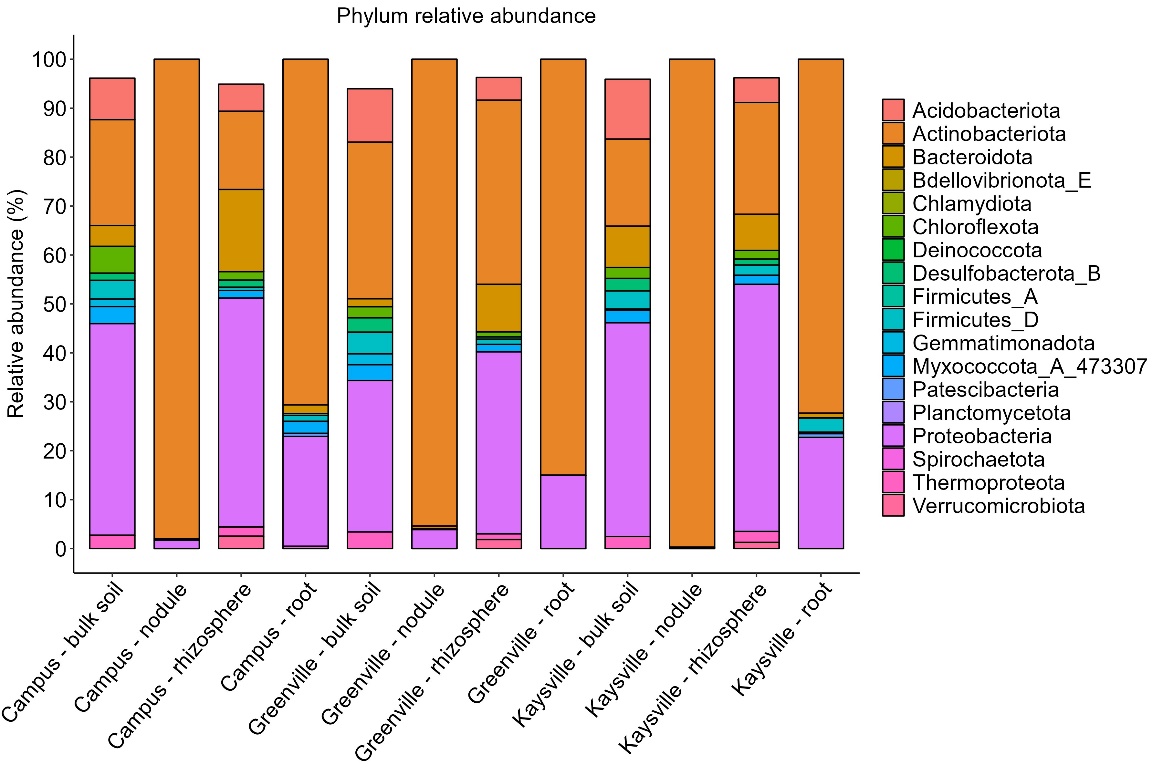


Figure S3 (A) Phylogenetic histogram at phylum level


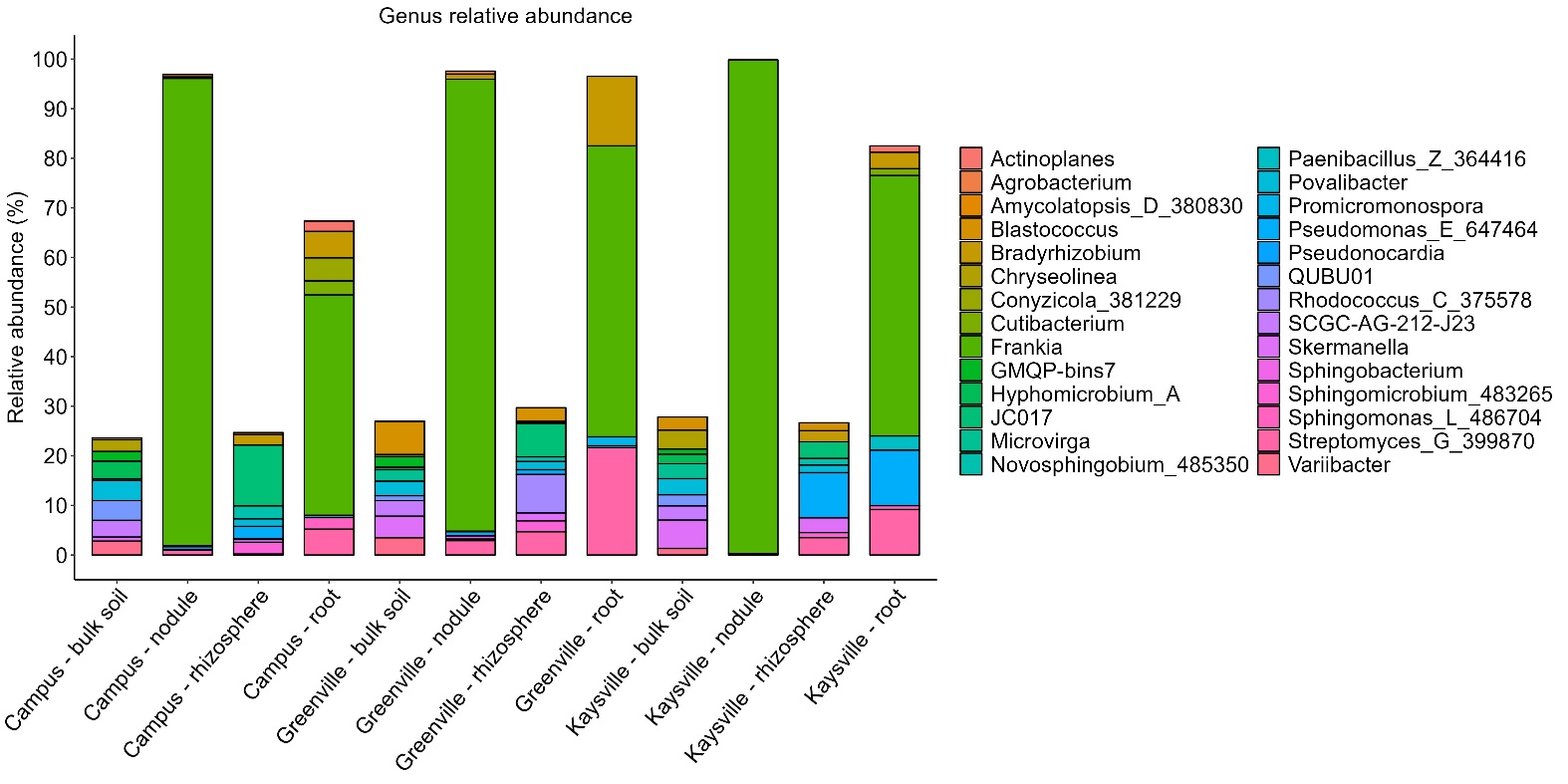


Figure S3 (B) Phylogenetic histogram at genus level

Figure S4 CCA plot


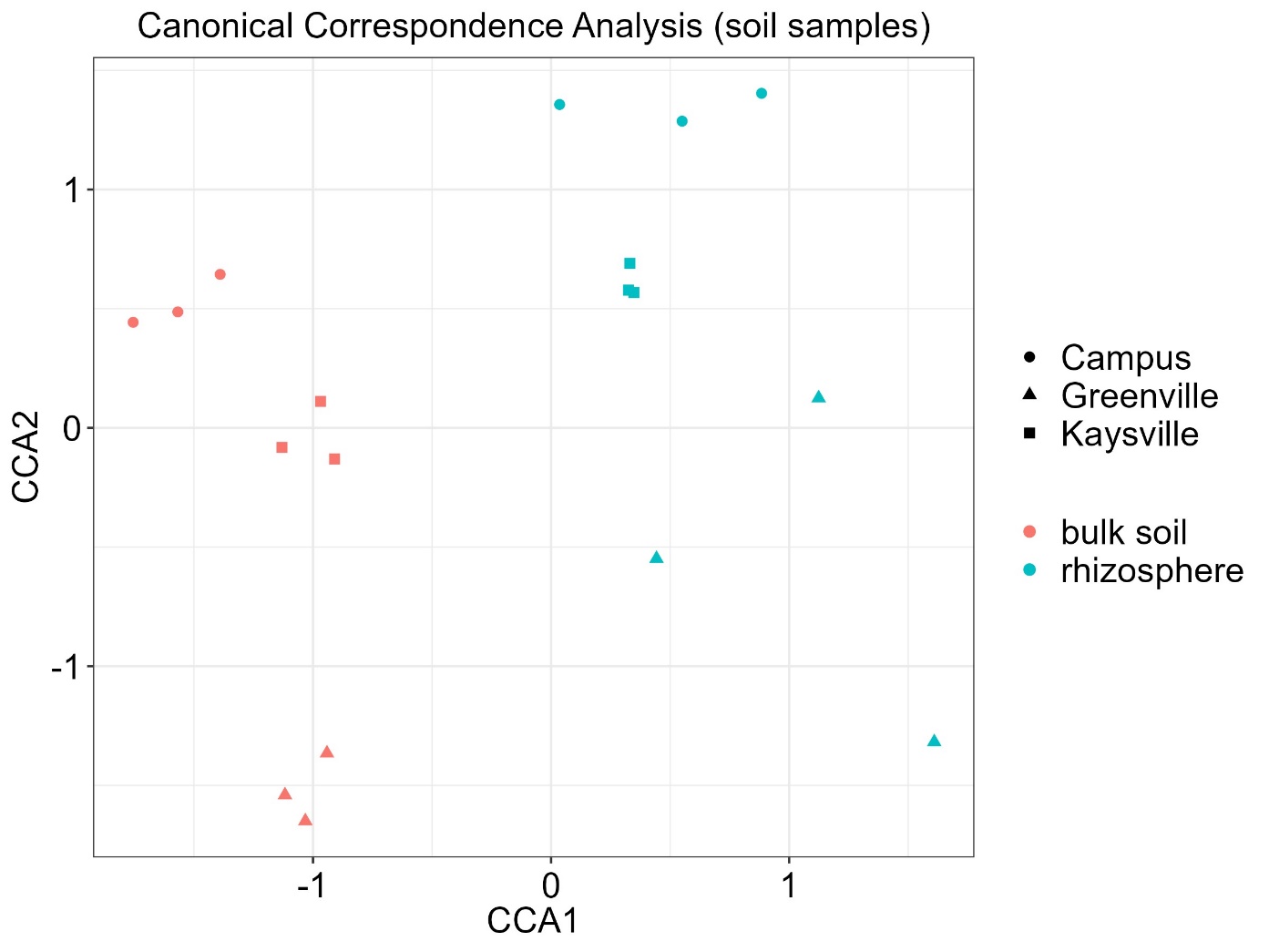


Figure S4 (A) CCA plot of bulk soil and rhizosphere samples of hybrid buffaloberry across three locations


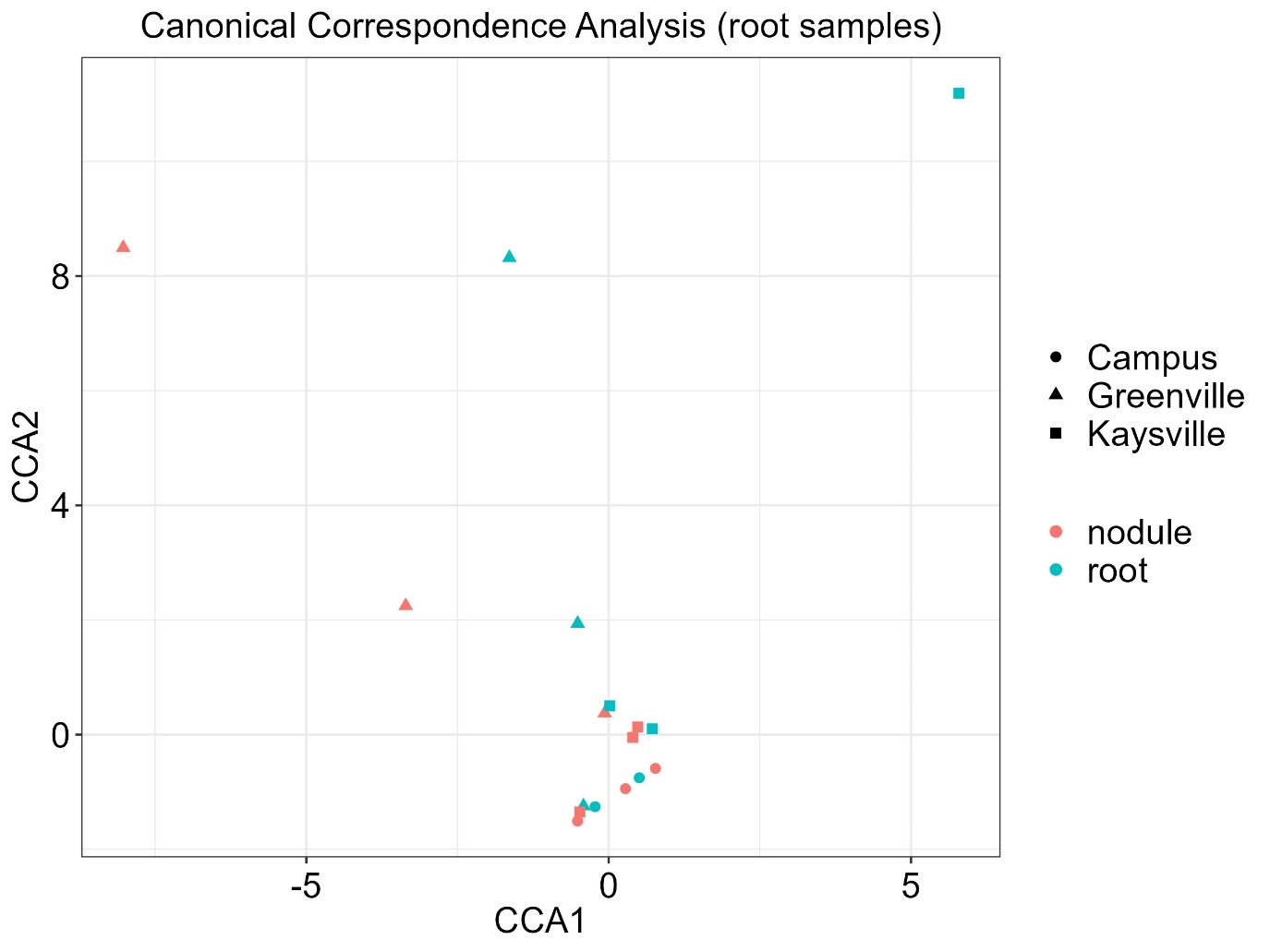


Figure S4 (B) CCA plot of root and nodule samples of hybrid buffaloberry across the three locations
